# Integrating word-form representations with global similarity computation in recognition memory

**DOI:** 10.3758/s13423-023-02402-2

**Published:** 2023-11-16

**Authors:** Adam F. Osth, Lyulei Zhang

**Affiliations:** University of Melbourne, Melbourne, Australia

**Keywords:** Recognition memory, Orthographic representations, Semantic space models, Linear ballistic accumulator

## Abstract

In recognition memory, retrieval is thought to occur by computing the global similarity of the probe to each of the studied items. However, to date, very few global similarity models have employed perceptual representations of words despite the fact that false recognition errors for perceptually similar words have consistently been observed. In this work, we integrate representations of letter strings from the reading literature with global similarity models. Specifically, we employed models of absolute letter position (slot codes and overlap models) and relative letter position (closed and open bigrams). Each of the representations was used to construct a global similarity model that made contact with responses and RTs at the individual word level using the linear ballistic accumulator (LBA) model (Brown & Heathcote *Cognitive Psychology*, *57* , 153–178, 2008). Relative position models were favored in three of the four datasets and parameter estimates suggested additional influence of the initial letters in the words. When semantic representations from the word2vec model were incorporated into the models, results indicated that orthographic representations were almost equally consequential as semantic representations in determining inter-item similarity and false recognition errors, which undermines previous suggestions that long-term memory is primarily driven by semantic representations. The model was able to modestly capture individual word variability in the false alarm rates, but there were limitations in capturing variability in the hit rates that suggest that the underlying representations require extension.

**Supplementary Information:**

The online version contains supplementary material available at 10.3758/s13423-023-02402-2.

Possibly the single most important cornerstone of theories of episodic memory is the encoding specificity principle (Tulving & Thomson, 1973), which states that retrieval of a given memory is proportional to the similarity between the memory-in-question and the cues present at the time of retrieval. This principle is contrary to early notions of forgetting, which placed considerably greater emphasis on the strength of the encoded memories or the amount of time between the learning and retrieval events. Since its inception, virtually all successful memory models embody this concept at the core of the theory, including models of recognition memory (Cox & Shiffrin, 2017; Dennis & Humphreys, 2001; Osth & Dennis, 2015; Shiffrin & Steyvers, 1997), free recall (Howard & Kahana, 2002a; Lehman & Malmberg, 2013; Polyn et al., 2009; Raaijmakers & Shiffrin, 1981), and serial recall (Farrell & Lewandowsky, 2002; Brown et al., 2000; Henson, 1998). In these models, both the successes and errors of retrieval are direct consequences of similarity – the test cues may be sufficiently dissimilar to a learned representation to prevent remembering, or spurious similarity between the test cues and learned information can result in a false memory of an item having been studied.

Placing similarity at the heart of retrieval does, however, invite a further question – what defines the similarity between cues and memories? Without a definition of similarity, such models risk falling prey to the circularity problem – the memory judgments themselves define the similarity between cues and studied representations with no recourse to an independent standard. While the precise definition of similarity is a very large question (e.g., Medin, Goldstone, & Gentner, 1993; Tversky, 1977), models of episodic memory often define similarity as the overlap between the representation of a probe and a stored representation in memory. However, precisely defining the content of such representations is challenging, as finding "true" psychological representations might be considered the holy grail of cognitive science more generally. Many models circumvent the problem by randomly generating stimulus representations – similarity effects can be captured by assuming that items from a common category share features (e.g., Criss & Shiffrin, 2004; Hintzman, 1988). While this allows models to make predictions about different categories or list conditions, there are no tractable means for making predictions about individual items in an experiment.

Fortunately, there has been progress in defining stimulus representations that allow models to make predictions with fewer parameters, and even make predictions on an item-by-item basis. In recent years, memory models have been able to capitalize on the successes of the semantic space models (see Günther, Rinaldi, & Marelli, 2019; Jones, Willits, & Dennis, 2015 for reviews). Using a large corpus of natural text, semantic space models learn representations of words from their co-occurrence with other words in the text. The resulting representations bear similarity relationships that resemble human judgments – the similarity between the representations of "boy" and "girl" will be higher than the similarity between unrelated concepts such as "truck" and "pool." Memory models have enjoyed some success in using semantic representations from such models as word representations in both recognition memory (Johns et al., 2012; Monaco et al., 2007; Osth et al., 2020; Reid & Jamieson, 2023; Steyvers, 2000) and recall tasks (Kimball et al., 2007; Mewhort et al., 2018; Morton & Polyn, 2016; Polyn et al., 2009). A distinct advantage of this approach is that false memory errors as a consequence of semantic similarity emerge "for free" – when a list of highly similar items is studied, the high degree of overlap between the representations of unstudied probe words and the list words naturally leads to the prediction of false memory errors.

However, few models have further specified perceptual representations of words, despite the fact that perceptual similarity among words produces the same types of false memory errors as semantic similarity (e.g., Shiffrin, Huber, & Marinelli, 1995; Sommers & Lewis, 1999; Steyvers, 2000; Watson, Balota, & Roediger, 2003). One notable exception is the dissertation of Steyvers (2000), who specified a variant of the retrieving effectively from memory (REM) model that employed semantic representations derived from word association spaces in addition to orthographic representations of the letter strings. Specifically, Steyvers employed what is referred to as a *slot* code – letters are coded with respect to their absolute position of occurrence, meaning that the letters in the word "cat" would be encoded as "c" in the first position, "a" in the second position, and "t" in the third position.

As we will elaborate below, there are a number of other representational schemes besides slot codes for representing letter position, each with their own consequences for similarity between letter strings. An additional class of representations are models that code for the relative position within a letter string, such as coding for whether letters are adjacent to other letters in the string or alternatively coding for the distances between letters in the string, without regard to their absolute position.

The present work aims to explore the consequences of such representations on both the accuracy and latency of episodic recognition memory decisions. While many models and analyses only consider the accuracy of recognition memory, the latency of recognition memory decisions often co-varies with accuracy such that more accurate stimuli or conditions often exhibit shorter response times (Murdock & Dufty, 1972; Ratcliff & Murdock, 1976). In the first part of the article, we will describe a set of perceptual representations of words that define string similarity solely in terms of letter position. While the similarity among the letters undoubtedly would play a role, the positional schemes we pursue in this work are relatively simple and can capture similarity effects in recognition memory, where orthographic similarity is often manipulated by the number of shared letters rather than the similarity of the letters themselves (e.g., Sommers & Lewis, 1999). These representations of letter order are quite common in the psycholinguistics literature and have often been used either as the core representations of models of reading (Coltheart et al., 2001; McClelland & Rumelhart, 1981; Snell et al., 2018) or to explain phenomena such as priming effects and response times in same-different judgments among pairs of letter strings (Davis & Bowers, 2006; Gomez et al., 2008). In this work, we will factorially explore representations that a.) code for absolute or relative position of the letters within the string and b.) code with respect to one other element or multiple elements within the string, and, for the class of absolute position models, c.) whether letter position is purely forward-ordered or whether there are both forward- and backward-ordered representations. To date, such representations have not been used to explore perceptual confusions between words in episodic memory tasks, although they do bear a resemblance to the representational schemes in models of serial order memory (Caplan, 2015; Osth & Hurlstone, 2023).

These orthographic representations will make contact with recognition memory data by calculation of the *global similarity* between the probe word and each of the words in memory – each probe-item similarity will be averaged together to produce an index of how similar the probe is to the contents of memory, as in global matching models of recognition memory (e.g., Clark & Gronlund, 1996; Osth & Dennis, 2020). The global similarity values will make contact with both choice and latency data from individual participants via the linear ballistic accumulator model (Brown & Heathcote, 2008), which enables the models to make contact with both choice and response time data simultaneously.

In the second part of the article, we will explore how orthographic representations combine with semantic representations, specifically those from Word2Vec (Mikolov et al., 2013). When both orthographic and semantic representations are included, we can measure the respective weights of both representations in determining the similarity between the probe and each memory, which allows us to measure how these weights vary across shallow and deep processing tasks in a depth of processing manipulation. In addition, the models will also be tested to evaluate how well they capture memory performance on an item-by-item basis in a recognition memory megastudy of individual words (Cortese et al., 2015).

## Orthographic representations

Orthographic representations are representations of the word form, specifically how the letters are arranged together. The similarity of such representations has clear consequences for episodic memory – when categories are constructed of orthographically similar words, such as *mate*, *late*, and *date*, one often finds the same patterns of performance as with semantic categories, namely an increase in the false alarm rate (FAR) as the category size on the study list is increased (Heathcote, 2003; Shiffrin et al., 1995; Steyvers, 2000), as well as high FAR to a non-presented prototype (Chang & Brainerd, 2021; Coane et al., 2021; Sommers & Lewis, 1999; Watson et al., 2003) when lists of orthographic/phonemic categories are constructed using the Deese-Roediger-McDermott (DRM) paradigm (Deese, 1959; Roediger & McDermott, 1995). In addition, lure probes that are orthographically similar to even a single target item often elicit higher rates of false recognition than lure probes than semantically similar lures (Cramer & Eagle, 1972; Gillund & Shiffrin, 1984). Finally, lure probes containing letters that were not present in any of the study list words are rejected substantially more easily than words that contain matching letters (Mewhort & Johns, 2000).

We will detail below a number of representational schemes for letter strings that can be used for each individual word in a recognition memory experiment. For each representational scheme, the matches between the memory and the probe string are summed together and divided by the alignment length, which is defined as the number of letters in the longer of the two letter strings, to produce a measure of similarity between 0 and 1.

In addition, each scheme contained weighting parameters that allowed for extra weight of the beginning and end letter of each string. This is on the basis of previous work in word identification that suggests that the exterior letters are more consequential for identification than the interior letters (Grainger & Jacobs, 1993; Jordan et al., 2003; Scaltritti et al., 2018; Whitney, 2001), which can be considered analogs of the primacy and recency effects at the within-word level.

### Absolute position codes

In the absolute position scheme, similarity between letter strings is highest when the letters occur in the same absolute position across the two strings. Absolute position can be coded relative to either the beginning or the end of the letter string. In the simplest schemes, a match is only considered if the letter occurs in the exact same position across the two strings. In variants based on the overlap model (Gomez et al., 2008), partial matches are allowed when a letter occurs in a similar position across the two strings. We will elaborate on these schemes below.

#### Slot codes

Slot codes involve the association of each letter within the word to its position relative to the start of the string. Words such as "candy" and "carton" can be recoded as $c_1a_2n_3d_4y_5$ and $c_1a_2r_3t_4o_5n_6$, which both share "c" in the first position and "a" in the second position. The similarity between the two strings is proportional to the number of these matches. Slot coding has traditionally been a very popular form of word-form representation. It was the representational scheme used by the interative activation model (McClelland & Rumelhart, 1981), the multiple readout model (MROM: Grainger & Jacobs, 1996), the dual-route cascade (DRC) model (Coltheart et al., 2001), as well as the connectionist dual process (CDP++: Perry, Ziegler, & Zorzi, 2010) model. In episodic memory, slot coding strongly resembles (Conrad, 1965)’s box model of serial order memory and was the representation of word-form used in (Steyvers, 2000)’s variant of the REM model.

Formally, we can express a match *m* on position *k* between two letter strings *i* and *j* as:

1 $$\begin{aligned} m_k = 1 \;\text {if}\; i_k = j_k\;\text {and}\;k \ne 1, k \ne j_{W(j)} \\ m_1 = \alpha \;\text {if}\; i_1 = j_1 \nonumber \\ m_{W(j)} = \Omega \;\text {if}\; i_{W(j)} = j_{W(j)} \nonumber \\ \text {otherwise}\; m_k = 0 \nonumber \end{aligned}$$

where *W* refers to the length of the word, $\alpha$ is a parameter for the weight of the match on the start letter, and $\Omega$ refers to the weight of the final letter. The $\alpha$ and $\Omega$ parameters are common to all of the models and are freely estimated to capture the importance of exterior letter effects. Here, we assumed that the $\Omega$ parameter only applies to the final letter of the studied word *j* and not the probe word. This assumption is inconsequential if both words are of the same length, but if the words are of different lengths, any changes in the weight of the terminal letter apply only to studied words and not to probed words.

The number of matches is simply the sum *m* across all letter positions:

2 $$\begin{aligned} M_{ij} = \sum _k{m_k} \end{aligned}$$

The similarity *s* between the two letter strings is:

3 $$\begin{aligned} s_{ij} = \frac{M_{ij}}{a_{ij} + (1 - \alpha ) + (1 - \omega )} \end{aligned}$$

where $a_{ij}$ is the alignment length of *i* and *j*, which is the length *W* of the longer of the two words. If the exterior letters are weighted the same as the other letters (e.g., $\alpha = 1$ and $\Omega = 1$), then the entire equation is simply the number of matches *M* divided by the alignment length *a*. The inclusion of $\alpha$ and $\Omega$ in the equation ensures that the similarity remains bounded between 0 and 1 as the weights of the exterior letters diverge from one.

According to the slot code scheme, the strings "baseball" and "based" would result in $M = 4$ due to "b", "a", "s", and "e" occurring in the same positions and $s = .5$ due to the division of *M* by 8 (the number of letters in "baseball") if all letters are equally weighted. The strings "raided" and "hamburger", in contrast, would result in $M = 1$ as there is only a single letter that occurs in the same position across the two strings ("a" in the second position).

Consequently, slot coding yields similar representations when words share the same stems. For instance, the words "breakfast" and "breaks" will exhibit high similarity due to sharing the first five letters in the same positions across the two words. However, while slot coding succeeds at aligning words relative to the beginning of the word, it is unable to align words that share the same suffixes or endings if the two words are of different lengths. For instance, the words "vacation" and "representation" share the same "-ation" ending, but the two words will exhibit little similarity under a slot coding representation due to the fact that the common letters are not aligned.

#### Both-edges slot codes

The fact that words of different lengths cannot be easily aligned within a slot code can be remedied by augmenting the representation with representations that are aligned to the ends of the words. This is referred to as *both-edges coding* (Fischer–Baum et al., 2010; Jacobs et al., 1998). In this scheme, letters are represented relative to not just the beginning of the word, but the end of the word as well. Both-edges schemes have been employed in competitive queuing models of spelling (Glasspool & Houghton, 2005) as well as the MROM-p model (Jacobs et al., 1998). In episodic memory, the start-end model (Henson, 1998) employs a both-edges scheme in that each item in a list is encoded relative to the beginning and end of the list.

We use the slot codes defined in the previous section to define the start-based code. The end-based code can be done in a similar manner by coding each position in the word relative to the end of the word. As an example, we could define the word "kitten" as $k_{-6}i_{-5}t_{-4}t_{-3}e_{-2}n_{-1}$ and "smitten" as $s_{-7}m_{-6}i_{-5}t_{-4}t_{-3}e_{-2}n_{-1}$. Using this positional scoring, we can calculate *m*, *M*, and *s* according to Eqs. 1, 2, and 3 above. These two strings share four letters, resulting in a similarity of 4/7 or .571. The start-based similarity, in contrast, is zero (0/7 matches). For the end-based slot code, the $\alpha$ and $\Omega$ parameters still correspond to the start and end of the initial string.

Overall similarity between two letter strings *i* and *j* in the both-edges code is a weighted combination of start-based and end-based similarities:

4 $$\begin{aligned} s_{ij} = ws_{ij,start} + (1-w)s_{ij,end} \end{aligned}$$

where *w* is estimated as a free parameter, but can reasonably be expected to be greater than .5 in most applications to data.

#### The overlap model

An alternative to both slot coding and both-edges representation is the overlap model (Gomez et al., 2008; Ratcliff, 1981). In the overlap model, rather than having letters associated with only a single position, letters are represented using positional uncertainty functions from the beginning of the string. The overlap model is often referred to as a "noisy" slot code because it is as if each letter occupies multiple letter position slots, but the strength is different for each position and the letter is most strongly associated to its true position. That is, consider the letter "d" in the word "badge" – the overlap model states that the "d" occupies all five letter slots, but is most strongly represented in the third position and is the weakest in the first and fifth positions. The positional uncertainty functions in the overlap model are analogous to the representations in many positional models of serial recall, where representations of within-list position overlap with each other (Brown et al., 2000; Burgess & Hitch, 1999; Lee & Estes, 1981), and also bear a resemblance to the CODE model of visual attention. (Logan, 1996).

The overlap model is formalized using Gaussian uncertainty functions – each letter is associated with a Gaussian distribution centered on its true position where the standard deviation of the Gaussian distribution is a free parameter and varies by serial position. In the overlap model, by convention the uncertainty is only present in one of the letter strings, which is usually either a prime or a briefly presented target. In the case of recognition memory, we define the uncertainty in the studied word, as this is only in memory and no longer presented to the participant. We will define *k* as the position of the letter in the probe string and *l* as the position of the same letter in the encoded string in memory:

5 $$\begin{aligned} m_k = G[\Phi (k + .5, l, \sigma = \sigma _l) - \Phi (k - .5, l, \sigma _l)];\text {if}\; i_k \in j \\ \text {otherwise}\; m_k = 0 \nonumber \\ G = \alpha \;\text {if}\; k = 1; G = \omega \;\text {if}\; k = W(j); \;\text {otherwise}\; G = 1 \nonumber \end{aligned}$$

where $\Phi$ is the cumulative distribution function of the normal distribution.

**Fig. 1:**
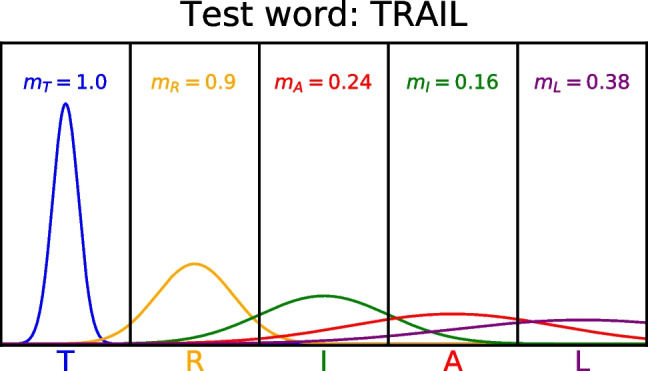
Illustration of the match calculation for two letter strings: a studied string “trial" in which each letter has uncertainty in its position coding, and a test string “trail." The match values *m* for each letter in the test string are also depicted. See the text for more details

$m_k$ is maximal when a letter occupies the same position in each word ($k = l$) and gets progressively weaker as *l* deviates from *k*. It is also important to note that *m* crucially depends on the standard deviation $\sigma$. As $\sigma$ approaches zero, the model reverts to the slot code as all of the probability density in the encoded letter is centered on its true position. However, as $\sigma$ increases, $m_k < 1$ even if $i_k = j_l$ because the probability density of the letter’s position is distributed across multiple letters. In other words, the overlap model as implemented by Gomez et al. (2008) makes no distinction between strength and uncertainty (Davis, 2010). To correct for this and to make the model comparable to the other models, we have introduced the $\alpha$ and $\Omega$ parameters, which allow for flexibly weighting the exterior letters without affecting the precision of the letter representations. These were applied in the same manner as the forward slot code model.

An illustration of the comparison between the studied string "trial" and the test word "trail" can be seen in Fig. 1. Each letter in the studied string is represented with uncertainty, which varies with letter position, and the higher uncertainty means that the letter’s identity "spills over" into nearby positions. The letter "T" is represented with high precision such that it is almost entirely concentrated in the first position, whereas the letter "L" is represented with low precision, such that there is a reasonable degree of probability mass in positions 3 and 4 of the string.

When the string "trail" is matched against the representation of "trial", the match values depend on both the displacement of the letters in the test string from the studied string and the degree of uncertainty in the studied letter. The match for the letter "T" is 1.0 because the probability density for "T" is entirely in the first position. If another test word was matched instead that contained the letter "T" in the second position instead, such as "atlas", the match on the letter "T" would instead be approximately zero because there is very little probability density of "T" in the second position.

One can also see that for the other matching letters, the match values are a function of the uncertainty of each letter position. The match value of "L" is quite low because the uncertainty is sufficiently high that there is little probability mass in its true position. Nonetheless, this match value is still higher than the mismatched letters "A" and "I", which have lower match values because each of these letters are displaced by one position and the probability mass for mismatching letters is lower than for matching letters.

Conventionally, the standard deviation parameter varies across each letter in the word, which can result in a very large number of free parameters, especially in recognition memory where words can be ten letters or longer. Fortunately, Gomez et al. (2008) found that the standard deviations could be described more simply as a two-parameter exponential function in which the standard deviation increases with letter position:

6 $$\begin{aligned} \sigma _l = d(1 - \text {exp}[-(i - .5)/r]) \end{aligned}$$

where *l* is the serial position of a letter in the word, *r* is the rate of growth, and *d* is the asymptote – both *r* and *d* are estimated as free parameters in all of our fits to data below.

#### Both-edges overlap model

While the overlap model can be considered a noisy slot code, it inherits some of the limitations of the slot code in that positions are represented relative to the start of the string. For this reason, we developed a both-edges overlap model that can be considered a noisy version of the both-edges slot code model described above. Equation 5 is used to calculate similarity, with the exception that backward similarity within the overlap model is calculated relative to the end of the string. The forward and backward similarities are combined using Eq. 4, which introduces the weighting parameter *w*.

### Relative position codes

In relative position codes, a letter is coded relative to other letters in the word rather than its position within the letter string. The most common implementation – and the one we pursue in this work – is coding the string in terms of adjacent letter bigrams. That is, a word such as "cat" can be decomposed into the bigrams CA and AT. The open bigrams allow for bigrams separated by other letters to be represented, such that an additional CT bigram would be represented in the string. While there are some models of word recognition that have considered letter trigrams (Seidenberg & McClelland, 1989), we know of little work exploring the consequences of these representations and have not pursued them in order to preserve a manageable number of representational schemes to explore.

#### Closed bigrams

The simplest relative position code involves coding the string into adjacent letter bigrams. Adjacent letter bigrams illustrate the advantage of relative position codes – similarity can be preserved if the absolute positions of substrings are perturbed across the two strings. For instance, consider the two words "members" and "remembering" – "member" is an embedded substring in both of these words, but there will be little alignment between the two strings according to absolute position schemes. If we break up the word into adjacent letter bigrams and use shared bigrams as the basis of similarity, we can see that both strings share the bigrams *me*, *em*, *mb*, *be*, and *er*. Closed bigrams resemble associative chaining models of serial recall, where items within a study list are directly associated to each other (Lewandowsky & Murdock, 1989). Direct associations between items are also common to address paired associate memory tasks and free recall (Criss & Shiffrin, 2005; Gillund & Shiffrin, 1984; Lehman & Malmberg, 2013; Osth, A. F., & Dennis, 2014; Osth & Dennis, 2015; Raaijmakers & Shiffrin, 1981). A disadvantage of all bigram schemes is that they do not have a natural way of capturing the importance of the exterior letters. For this reason, we augment the bigrams with representations of the beginning and end of the word. That is, the word "member" would also include $\_ m$ and $r \_$, where _ indicates the letter is an exterior letter, with the first position indicating that it is the first letter and the second position indicating that it’s the final letter. This is consistent with previous bigram representations (e.g., Hannagan & Grainger, 2012; Seidenberg & McClelland, 1989) and is also consistent with chaining models of serial order memory that additionally include representations of the start- and end-of-the-list (e.g., Lewandowsky & Murdock, 1989; Solway, Murdock, & Kahana, 2012).

A match *m* on a bigram *k* between strings *i* and *j* is scored as:

7 $$\begin{aligned} m_k = G \;\text {if}\; k \in i_\kappa \cup j_\kappa \\ \text {otherwise}\; m_k = 0 \nonumber \\ G = \alpha \;\text {if}\;k = 1; \;G = \omega \;\text {if}\;k = w(j); \text {otherwise}\; G = 1 \nonumber \end{aligned}$$

where the subscript $\kappa$ refers to the set of bigrams in the string. A complication with bigram coding that we found is that a bigram may be repeated in one word but not another. That is, consider if the string "ababab" is compared against the string "ab" – a similarity value of .50 can be returned if each "ab" in the first string can be matched against the bigram in the second. For this reason, we made it such that each bigram can only match another bigram in a comparison string once. This was accomplished by removing each matched bigram from the set of possible bigrams during the comparison process^1^. Evidence for this assumption comes from the fact that strings with matching repeated letters do not show any priming advantage over strings with non-repeated letters (Schooenbaert & Grainger, 2004).

The overall similarity *M* is calculated in the same manner as the other schemes (according to Eq. 3) but with the exception that the alignment length *a* refers to the number of bigrams in the longest of the two strings.

#### Open bigrams

Open bigrams make allowance for adjacent letters that are separated by other letters. To prevent an explosion of possible bigrams as word length increases, we follow Grainger and van Heuven (2003) and only allow bigrams between pairs of letters that have at most two letters between them. To give an example, if we considered all bigrams that could be constructed from the letter "a" in the word *about*, the closed bigram representation would only include the bigram "ab", while the open bigram representation would additionally include "ao" and "au." Open bigrams can be considered the relative position analog of the overlap model – just as the overlap model could be considered a "noisy" slot code, open bigrams could be considered a "noisy" bigram representation. However, open bigrams differ from the overlap model because the longer-range bigrams are conventionally weighted the same as the bigrams formed from adjacent pairs of letters. Open bigrams are employed in models of word recognition such as the SERIOL model (Whitney, 2001) as well as the OB1-Reader (Snell et al., 2018). In episodic memory, open bigram representations strongly resemble associations from models that employ remote associations, where associations are formed between items that are more than one item apart (Logan, 2021; Murdock, 1995; Solway et al., 2012).

The match *m* on a bigram *k* is calculated according to Eq. 7 except that the set of bigrams in each string is larger, which likewise affects the alignment length *a*.

### Levenshtein distance

We will be comparing each of the above representations to string similarity based on Levenshstein distance (Levenshtein, 1966). Levenshtein distance is a measure of string edit distance and refers to the minimum number of transformations (substitutions, insertions, or deletions) between any two strings. For instance, there is a Levenshtein distance of 1 between "dog" and "dogs" – this is because only a single operation (insertion of "s" into "dog" or removal of "s" from "dogs") is required to transform one string into another. Levenshtein distance has been used in various psychological applications, including quantification of orthographic density in the lexicon (Yarkoni et al., 2008), orthographic similarity effects in recognition memory (Freeman et al., 2010; Zhou et al., 2023), and measuring the difference between recalled sequences and study lists (Logan, 2021).

We can convert the Levenshtein distance between two strings $D_{ij}$ to similarity using the following equation:

8 $$\begin{aligned} s_{ij} = \frac{a_{ij} - D_{ij}}{a_{ij}} \end{aligned}$$

where the alignment length *a* refers to the number of letters in the longer of the two strings.

While Levenshtein distance is an extremely useful measure for computing string similarity, it was not designed as a psychological measure of orthographic similarity. For instance, each of the transformations that it considers are all equally weighted in the similarity calculation. In practice, each possible transformation often exhibits different consequences for perceived string similarity in masked priming tasks (Davis & Bowers, 2006; Hannagan & Grainger, 2012). While it would be possible to estimate different weights of these transformations, the psychological representations not only produce different weights and consequences for each operation, but they also provide more principled explanations for these phenomena that can be linked to associative mechanisms.

### Nearest neighbors

To illustrate the differences between the different orthographic representations, Table 1 shows the five *nearest neighbors* – the words with the highest similarity values – to three different words: *ledge*, *sustain*, and *yourselves*. Rather than calculate the similarity of these words to the entire lexicon, we used the word set from Cortese et al. (2015) – one of the datasets we fit – as a way to demonstrate similarity to potential memory set items. This dataset contained words that were between 3 and 10 letters long.

One should note that the similarities in each of these schemes depend on the values of their parameters. We fixed the values of $\alpha$ and $\Omega$ to 1 such that the start and end letters exhibited the same degree of importance as the other letters. If such parameters are increased, mismatches on these letters become much more consequential, ensuring that the nearest neighbors are much more likely to include words that match on these letters. For the both-edges slot and overlap models, we fixed the weight of the forward representation to .75. For the overlap models, we fixed the parameters of the exponential function to the best fitting values from Gomez et al. (2008), namely $r = 1.094$ and $d = 1.544$. In our fits to recognition memory data, we estimate the values of each of these parameters.

**Table 1:** Nearest neighbors to a set of four words from each orthographic representation

Rep.	Neighbors
*ledge*	
Slot	ledger (.83), ladle (.6), wedgie (.5), redeem (.5), midget (.5)
Both-edges slot	ledger (.62), ladle (.6), wedgie (.42), ladder (.42), venue (.4)
Overlap	ledger (0.3), lever (0.26), level (0.26), leper (0.26), legend (0.26)
Both-edges overlap	ledger (0.28), lever (0.24), level (0.24), leper (0.24), ladle (0.24)
Closed bigrams	ledger (.71), ladle (.5), knowledge (.5), wedgie (.43), peddle (.43)
Open bigrams	ledger (.71), legend (.5), ladle (.45), wedgie (.43), knowledge (.43)
Levenshtein	ledger (.83), wedgie (.67), ladle (.6), mileage (.57), knowledge (.56)
*sustain*	
Slot	curtain (0.71), sultan (0.57), suction (0.57), mustard (0.57), disdain (0.57)
Both-edges slot	curtain (0.71), suction (0.57), mustard (0.57), disdain (0.57), custard (0.57)
Overlap	session (0.25), sultan (0.24), suction (0.24), suspend (0.22), sushi (0.22)
Both-edges overlap	session (0.25), sultan (0.24), suction (0.24), station (0.23), suspend (0.21)
Closed bigrams	sultan (0.5), status (0.5), station (0.5), retain (0.5), obtain (0.5)
Open bigrams	station (0.59), satin (0.59), sultan (0.53), curtain (0.53), suction (0.47)
Levenshtein	sultan (0.71), curtain (0.71), restrain (0.62), mountain (0.62), fountain (0.62)
*brittle*	
Slot	wrinkle (0.57), wrestle (0.57), whistle (0.57), shuttle (0.57), scuttle (0.57)
Both-edges slot	wrinkle (0.57), wrestle (0.57), whistle (0.57), shuttle (0.57), scuttle (0.57)
Overlap	brothel (0.26), bottle (0.26), battle (0.26), critter (0.25), bitter (0.25)
Both-edges overlap	bottle (0.27), battle (0.27), critter (0.25), brothel (0.25), bitter (0.25)
Closed Bigrams	little (0.62), bottle (0.62), battle (0.62), title (0.5), shuttle (0.5)
Open bigrams	little (0.59), rattle (0.53), bottle (0.53), battle (0.53), title (0.47)
Levenshtein	rattle (0.71), little (0.71), bottle (0.71), battle (0.71), throttle (0.62)
*yourself*	
Slot	counsel (0.62), journey (0.5), journal (0.5), gourmet (0.5), voucher (0.38)
Both-edges Slot	counsel (0.47), journey (0.38), journal (0.38), gourmet (0.38), countess (0.38)
Overlap	yogurt (0.17), counsel (0.17), yonder (0.16), yodel (0.15), surreal (0.15)
Both-edges overlap	counsel (0.16), surreal (0.15), quarrel (0.15), yogurt (0.14), trousers (0.14)
Closed bigrams	recourse (0.44), myself (0.44), itself (0.44), yogurt (0.33), yodel (0.33)
Open bigrams	recourse (0.45), morsel (0.4), trousers (0.35), myself (0.35), itself (0.35)
Levenshtein	morsel (0.62), counsel (0.62), yodel (0.5), myself (0.5), journey (0.5)

Notes: Rep. = representation

The similarity is contained in parentheses

The words were sampled from the word set in the experiments of Cortese et al. (2015)

For the first word – "ledge" – each of the representations agree on the most similar word, which is "ledger." There is likely very broad agreement among the representations when two strings differ by an addition at the end of the word or a single substitution. One exception is that the bigram models predict lower values of similarity for this comparison. This is because while the absolute position schemes result in only a single letter difference between the two strings, the bigram models result in the mismatch of at least two bigrams – the "er" and "r_" bigrams.

Not all of the words have agreement among the nearest neighbors, however. For instance, the word "brittle" illustrates the difference between the absolute position and relative position schemes. The relative position schemes select the word "little" as their most similar word. The absolute position schemes selected different words – the slot-based models instead select "wrinkle", which is likely because "little" is one letter shorter than "brittle", which offsets the alignment of their absolute positions. The overlap model instead selects "brothel" while the both-edges overlap model selects "bottle" as the most similar word.

## Global similarity of orthographic representations

Each of the discussed orthographic representation schemes were used to predict performance on individual trials in four different recognition memory datasets. As mentioned previously, in recognition memory it is believed that recognition operates by computation of the global similarity – the similarity between the probe item and each of the learned representations from the study list is computed and aggregated together. The global similarity value is then subjected to a decision process to produce an "old" or "new" decision. While models such as REM and Minerva 2 produce their similarities by randomly generating representations of each list word, in this work the similarities are supplied by each of the representational schemes. Specifically, we implement each representation as its own global similarity model.

**Fig. 2:**
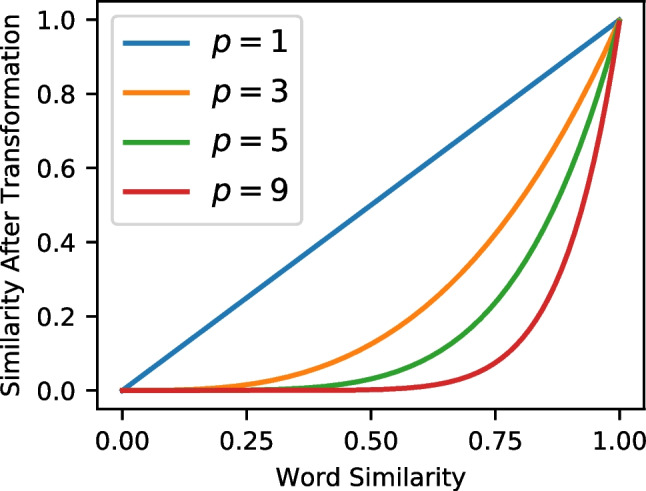
Similarity before (*x*-axis) and after (*y*-axis) the power transformation for four different values of *p* (1, 3, 5, and 9). See the text for details

**Fig. 3:**
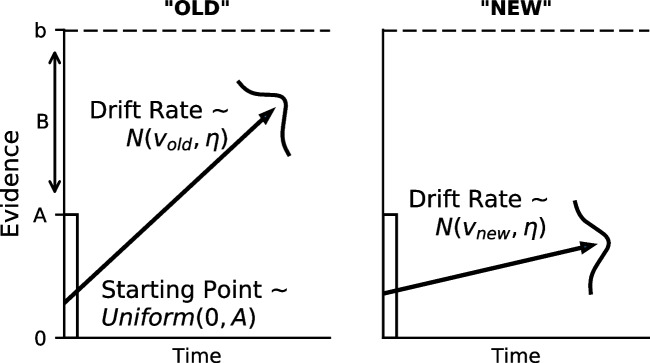
The linear ballistic accumulator (LBA). See the text for more details

The global similarity *g* for a probe word *i* can be computed as follows:

9 $$\begin{aligned} g_i = (\sum \limits _{j \in L j \ne i}s_{ij}^p) / N_L \end{aligned}$$

where *L* is the set of study list words, $N_L$ is the length of the study list, and *p* is a freely estimated non-linearity parameter. Similar to our previous investigation on semantic similarity (Osth et al., 2020), we omitted the target item from the global similarity computation. This is because in the majority of the orthographic representations, the self-similarity is always equal to 1. By omitting the self-similarity parameter, the investigation is more directly focused on how the *inter-item* similarity affects recognition memory decisions.

The non-linearity parameter *p* was directly inspired by the cubic transformation of similarities in the Minerva 2 model (Hintzman, 1988), which has the effect of punishing low similarity values before computing the global similarity. Because similarity is bounded between 0 and 1, there are no sign reversals for certain values of *p* (e.g., negative similarities becoming positive when *p* is an even number). The consequences of this transformation can be seen in Fig. 2. As the *p* parameter is increased, lower values of similarity are pushed to zero while higher values are more resistant, with similarity values of 1.0 being completely unaffected by the transformation. This non-linearity is also analogous to the *c* parameter in variants of the generalized context model (Nosofsky, 1986; Nosofsky et al., 2011). A psychological interpretation of this transformation is that it approximates auto-associative bindings, which are essentially bindings of stimulus features to each other – a mathematical analysis of the cubing process in the Minerva 2 model found that it was identical to a mode-4 autoassociative tensor (Kelly et al., 2017).

Arndt and Hirshman (1998) demonstrated that the cubing process in Minerva 2 was essential in capturing many phenomena in the false memory literature. We will demonstrate below that it was quite crucial in our fits to recognition memory data with orthographic representations for two reasons. First, highly similar lures exhibited much higher rates of false recognition than lures of moderate or low similarity to a much larger degree than a linear model predicted (a model where $p = 1$). Second, we will demonstrate that this parameter has the effect of reducing the similarity of the majority of memory set items to zero, such that only a small number of items that are similar to the probe contribute to the global similarity. In other words, this parameter functions to reduce the noise in the comparisons to the memory set.

## Mapping global similarity to old-new decisions: the linear ballistic accumulator (LBA) model

To make contact with experimental data, global similarity has to be mapped to old-new decisions. Many models accomplish this by comparing the global similarity to a response criterion, with responses above the criterion eliciting "old" responses (e.g., Gillund & Shiffrin, 1984; Hintzman, 1988). However, it is becoming increasingly common to use global similarity to drive an evidence accumulation process of decision-making, which is able to produce a decision but can additionally make predictions about the latency with which decisions are made (Cox & Shiffrin, 2017; Fox et al., 2020; Nosofsky et al., 2011; Osth et al., 2018).

In this work, we followed the evidence accumulation approach and used the linear ballistic accumulator (LBA) model (Brown & Heathcote, 2008). In the LBA (depicted in Fig. 3), each response alternative is associated with its own accumulator, which race to produce a decision. Each accumulator has a mean drift rate *v*, which determines the average slope of the accumulator – higher drift rates produce steeper slopes, and hence faster evidence accumulation. The decision ends when an accumulator’s response threshold *b* is reached, which not only produces the corresponding decision, but also the response time, which is the sum of the time elapsed during evidence accumulation plus some additional time $t_0$ for non-decision processes, such as processing of the stimulus and response execution.

A defining feature of the LBA is that evidence accumulation is both linear and noiseless. The stochastic aspects of the model instead come from between-trial variability in the model parameters. The starting point for evidence accumulation is sampled from a uniform distribution with height *A*, while the trial’s drift rate is sampled from a normal distribution with standard deviation $\eta$^2^. The between-trial variability parameters additionally function to allow for the prediction of fast errors under speed emphasis and slow errors under accuracy emphasis, similar to their roles in the diffusion decision model (Ratcliff & McKoon, 2008).

The threshold parameter *b* is responsible for the speed–accuracy tradeoff: increases in *b* slow decisions because the accumulators have to travel more distance to hit the threshold, but the decisions are more accurate because there is more time to overcome the noise in the starting point variation. In addition, bias can be accommodated in the model by allowing different thresholds for each response option. In this work, we estimate the *B* parameter, which is the distance from the top of the starting point distribution to the threshold *b* ($b = A + B$) and allow for bias by having two threshold parameters $B_{\text {old}}$ and $B_{\text {new}}$.

In this work, we map the global similarity *g* for a probe stimulus *i* in experimental condition *m* and old-new status *n* of the probe stimulus (e.g., target or lure) mean drift rates $v_{\text {old}}$ and $v_{\text {new}}$ using the following equations:

10 $$\begin{aligned} v_{\text {old},imn}&= V_0 + V_{mn} + \gamma _{mn}g_{im} \end{aligned}$$

11 $$\begin{aligned} v_{\text {new},imn}&= V_0 - (V_{mn} + \gamma _{mn}g_{im}) \end{aligned}$$

where $V_0$ is a freely estimated shift parameter that helps ensure that the drift rates are positive (e.g., van Ravenzwaaij et al., 2020), *V* is a mean drift rate that can vary across experimental conditions *m* or old-new status *n*, and $\gamma$ is a scale parameter that maps global similarity to drift rates. One can see from Eqs. 10 and 11 that increases in global similarity *g* serve to increase the mean drift rate $v_{\text {old}}$ and simultaneously decrease the drift rate $v_{\text {new}}$, resulting in more frequent "old" decisions that are faster. This parameterization mirrors the changes in drift rates in the diffusion decision model, where changing the strength of evidence for one response necessarily decreases the strength of evidence for the other. To insure identifiability we fixed the $\eta$ parameter to 1 for lures but freely estimated the parameter for target items ($\eta _{\text {target}}$), as previous investigations have found evidence of greater drift rate variability for target items (e.g., Osth et al., 2017; Starns & Ratcliff, 2014).

The purpose for including the *V* parameters is that in several datasets, we fit manipulations that alter performance – such as word frequency, depth of processing, and repetitions – for which global orthographic similarity may be able to partially explain but cannot provide a sufficient account of the changes of performance on its own. By allowing the *V* parameter to vary across conditions, it allows us to capture changes in performance across these conditions while being agnostic to the causes of these effects.

Essentially, Eqs. 10 and 11 can be considered regression equations where the global similarity is mapped onto the mean drift rates on a trial-by-trial basis. We allow the $\gamma$ parameter to vary across targets and lures because it allows us to measure the extent to which global similarity may influence targets and lures in different ways. For instance, in previous work using a similar approach with global semantic similarity, Osth et al. (2020) found that global similarity substantially affected drift rates for lures while producing only minimal effects on targets. While process models are unable to map global similarity to targets and lures in different ways, they are often able to mimic the finding that inter-item similarity has less of an effect on targets. One such example is the REM model (Shiffrin & Steyvers, 1997), where the self-similarity of a probe to its own representation in memory is both large and highly skewed, allowing it to dominate the global similarity computation such that the similarity of the probe to other items in memory would not substantially affect the resulting global similarity.

## The model fit

### Applying the models to data

Each representational scheme – the slot code, the both-edges slot code, the overlap model, the both-edges overlap model, closed bigrams, open bigrams, and Levenshtein distances – were all applied as separate global similarity LBA models to each of the four datasets. Within each dataset, we fit the models to each individual response and response time (RT) using hierarchical Bayesian methods (see Boehm et al. 2018), where parameters for individual participants and the group are jointly estimated. This avoids averaging artifacts associated with fitting group data (e.g., Estes & Maddox, 2005) but simultaneously allows for "pooling" information across individuals because each participant’s parameter estimates are influenced by the group-level parameters. Parameters were estimated using differential evolution Markov chain Monte Carlo (DE-MCMC), which is advantageous for fitting models like the LBA due to the presence of strong correlations between the parameters of the model (Turner et al., 2013).

For each response, the likelihood of the response and RT were computed according to the LBA’s likelihood function using the estimated parameters, including the mean drift rates for the individual trial that were computed based on the global similarity of the probe to each of the studied strings. Higher likelihoods reflect closer correspondences between the data and the model’s predictions. In hierarchical Bayesian models, the likelihood of a participant’s data under that participant’s model parameters is multiplied by the likelihood of those parameters under the group-level distribution. This is how the "pooling" occurs – participant parameters that are closer to the group level are more likely, and the resulting parameter estimates strike a balance between a good fit of the participant parameters and the correspondence with the group. Group-level distributions were either normal distributions or truncated normal distributions to capture the effects of boundaries, each with parameters $\mu$ and $\sigma$. Throughout the article, we denote the group-level parameters using superscripts (e.g., $A^\mu$ refers to the group mean of the *A* parameter). Additional details on the prior distributions and the MCMC sampling can be found in the Appendix.

Fitting these models to data was challenging and extremely lengthy in some cases. Each trial required aggregating the similarity of the probe to the $N_L$ items, where $N_L$ is the number of items on the study list. With the overlap model, the similarity between two words required calculating the match value on the individual letters. For the overlap model, this meant that each participant required the calculation of the number of observations *O* multiplied by $N_L$ list items multiplied by at most the longest length of the word. For our largest dataset, this meant that each participant required around 1.47 million calculations for the application of the overlap model, and this was doubled for the both-edges overlap model. Similarities to each item could not be pre-computed unless the parameters governing the representations and the non-linearity parameter *p* were fixed.

Each of the models varies in their number of parameters and their resulting complexity. For instance, the forward slot code can be considered a special case of the overlap model where all of the standard deviation parameters are set to zero, and also a special case of the both-edges slot code with the weight entirely on the start-based representation. Thus, the overlap model should inherently fit better than the slot code model. We make comparisons between each model using model selection techniques, which subtract a measure of model complexity from each model’s measure of goodness-of-fit. Specifically, we use the widely applicable information criterion (WAIC:Watanabe, 2010). WAIC is an approximation to leave-one-out cross validation. Because WAIC is on a deviance scale, lower values are preferred. In contrast to the deviance information criterion (DIC), WAIC is considered to be a "fully Bayesian" information criterion in the sense that its penalty term is determined by calculation of the variability in the likelihood of each data point across all of the parameters in the posterior distribution. In other words, a more complex model has more "ways" it can fit the data than a less complex model. WAIC has been recommended over DIC (Gelman et al., 2014).

### Datasets

We specifically employed recognition memory datasets with lists composed of unrelated words that do not contain any obvious similarity structure, similar to past approaches exploring the consequences of semantic similarity in free recall (Howard & Kahana, 2002; Morton & Polyn, 2016) and recognition memory (Osth et al., 2020). While several studies have explored the consequences of studying categories of orthographically similar words, the majority of these studies have done so by constructing a set of words with only 1-2 letters or phonemes difference from each other (e.g., Shiffrin et al., 1995; Sommers & Lewis, 1999). We find lists composed of highly similar items undesirable for two reasons. First, when studying a list of highly similar words like "mate", "late", "date", etc., it is possible that participants adopt different encoding or retrieval strategies, such as placing more emphasis on encoding common features or evaluating test items to determine whether they contain the common features. Second, we have established in Table 1 that each of the orthographic representations would likely agree that such items are highly similar to each other. Instead, there was more disagreement among the moderate similarity items, which should be more prominent in lists of unrelated words.

A total of four datasets were included in our fits to data, which are summarized in Table 2. The datasets vary considerably in their list lengths (ranging from 40 to 150 words), word lengths (3–11 letters), and experimental manipulations (word frequency, speed–accuracy emphasis, numbers of presentations, and levels of processing). The differences in list length among the datasets are consequential when one considers that global similarity always involves an average of all similarities in the word set. With longer lists, more similarity values contribute to the global similarity calculation. Details on exclusions from the data can be found in the Appendix. The raw data can be found on our OSF repository (https://osf.io/hyt67/).

**Table 2:** Summary of the datasets fit by the model

Dataset	$N_P$	*O*	$N_L$	$N_W$	$W_L$	Manipulation
Rae et al. (2014)	47	757	40	1612	5.1 (4-7)	SA emphasis, WF (LF/HF mixed)
Criss (2010, E2)	16	1412.9	50	1600	5.9 (4-11)	Pres. (1x/5x), WF (LF/HF crosslist)
Kiliç et al. (2017, E1)	30	1736.8	150	2929	5.9 (4-8)	DOP (deep/shallow)
Cortese et al. (2015)	119	2947.6	50	3000	6.2 (3-10)	None

Notes: E = experiment, $N_P$ = number of participants, *O* = mean number of observations per participant, $N_L$ = study list length, $N_W$ = number of words in the word set, $W_L$ = word length (mean, min-max). WF = word frequency, SA = speed–accuracy, Pres. = number of presentations, LF = low frequency, HF = high frequency, DOP = depth of processing

The data from Cortese et al. (2015) are noteworthy because all participants in the sample were tested on the same set of 3000 words. The large number of participants ($N = 119$) meant that there was a substantial number of observations for each individual item. Because global similarity varies on an item-by-item basis, it allows for us to test how each representational scheme is able to account for the variability in performance among the individual words.

For each of the datasets – with the exception of the Kiliç et al. dataset – the parameters governing the orthographic representations were all fixed across each of the conditions in the experiment. We did, however, allow several parameters within the LBA to vary across conditions. For the speed–accuracy emphasis manipulation in the Rae et al. (2014) dataset, we followed the original authors and allowed boundary separation *B*, starting point variability *A*, nondecision time $t_0$ and drift rate *V* to vary across the conditions. For the word frequency manipulation in the Criss and Rae et al. datasets, the drift rate *V* varied across conditions for both targets and lures. For the Criss dataset, drift rate *V* was varied across the repetition conditions for both targets and lures because it was a cross-list strength manipulation, which resulted in reduced FAR in the lists with repeated items. Indeed, the diffusion model analysis of Criss (2010) on the same dataset confirmed that the reduced FAR were due to changes in drift rates for the lure items. Each of these manipulations, however, were not the focus of the present work. A complete list of base LBA parameters common to all models can be found in the Appendix.

#### Allowances for depth of processing

**Fig. 4:**
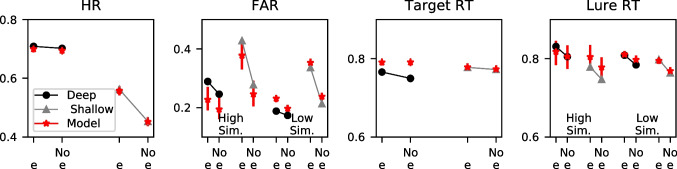
Group-averaged hit rates (HR), false alarm rates (FAR), mean RT to targets, and mean RT to lures, for probe items that both contain and lack the letter "e". Depicted are both the data and the posterior predictives of the selected model. *Error bars* depict the 95% highest density interval (HDI)

We made an exception on fixing the parameters governing the orthographic representations for the data of Kiliç et al. (2017), which used a depth of processing manipulation. In each study list, participants were either instructed to engage in shallow processing, which involved deciding whether an "e" was in the letter string, or were instructed to engage in deep processing, which involved deciding whether each word had a pleasant meaning. Their studies found that the deep processing task elicited better performance, including a reduced false alarm rate (FAR). An unexpected finding from this dataset was that hit and false alarm rates were both higher for probes containing the letter "e," a tendency that was especially pronounced in the shallow processing condition. These results can be seen in Fig. 4, which also separately show the results for high similarity lures (lures with a Levenshtein distance of 1 to one of the studied items) and lower similarity lures (lures with a minimum Levenshtein distance of 2 or higher to each of the studied items).

None of the representations could naturally capture this finding, so we used a modified inter-item similarity $s^*$ to take into account whether an "e" was present in both the probe and the stored representation:

12 $$\begin{aligned} s^{*}_{ij} = (M + E) / (a + (1 - \alpha ) + (1 - \omega ) + \epsilon ) \end{aligned}$$

13 $$\begin{aligned} E = \epsilon ;\text {if}\; \text {'e'} \in i \cup j ;\text {otherwise}\; E = 0 \end{aligned}$$

where $\epsilon$ is the strength of the letter "e". One should note here that the presence of the letter "e" in both strings increases the string similarity regardless of the position of "e" in either of the two strings. We used this approach because the shallow processing condition only instructs the participant to find an "e" in the word and does not specify that the participant reports its position. Figure 4 shows the predictions of the selected model (see the next section for details), which reveal that the model was able to capture the higher tendency to respond "old" to items that contain the letter "e."

**Table 3:** $\Delta$ WAIC values for each model variant in each dataset

	Criss		Rae		Cortese		Kiliç	
Model	$\Delta$ WAIC	*P*	$\Delta$ WAIC	*P*	$\Delta$ WAIC	*P*	$\Delta$ WAIC	*P*
Slot	101	21	32	23	508	13	136	24
Both edges	110	22	27	24	426	14	94	25
Overlap	79	23	33	25	828	15	**0**	26
Both-edges overlap	101	26	45	28	854	18	13	29
Closed bigrams	**0**	21	1	23	120	13	135	24
Open bigrams	74	21	**0**	23	**0**	13	137	24
Levenshtein	55	19	17	21	982	11	257	20

In addition, we found better performance of the models when other parameters relating to inter-item similarity changed across conditions, namely the $\alpha$, $\Omega$, *p*, and $\gamma$ parameters. For the overlap model, however, we fixed the *d* and *r* parameters across conditions as allowing such parameters to vary compromised identifiability and did not appreciably improve the fit of the model.

### Model selection

WAIC values for each model in each dataset can be seen in Table 3. One can see that there is no clear winner across all of the datasets – closed bigrams are preferred for the Criss (2010) dataset while open bigrams are preferred for the Rae et al. and Cortese et al. datasets. While this may seem as if relative position representations should be preferred overall, the Kiliç et al. dataset shows a preference for the overlap model. While it is difficult to ascertain the causes of this discrepancy, one possibility is that the encoding tasks that are part of the depth-of-processing manipulation in the Kiliç et al. dataset changed the way in which participants represented the letter strings.

In addition, we would also like to mention that the data of Cortese et al. are especially diagnostic due to the especially large size of that dataset. Table 3 also reveals that the WAIC scores between the selected model (open bigrams) and the other models is very large. As we will later discuss, this dataset was also noteworthy because several of the absolute position models failed to capture the differences in the false alarm rate between high and low similarity lures, which was not a problem for the relative position models.

One clear consistency was that models based entirely on Levenshtein distances were not preferred in any of the datasets, suggesting that there are clear advantages for considering psychological representations in capturing orthographic similarity effects.

As we will discuss shortly, there are many difficulties in adjudicating between the models that come from the nature of the recognition memory paradigm – having $N_L$ items on the study list means there are $N_L$ comparisons on any given trial, so it can be unclear which words from the study list contributed to a given response on a given trial. This is made much simpler in a priming task, where it can be more safely assumed that the most recently presented item had the largest contribution to performance on a given trial.

In the coming sections, we will first give an example of the similarity values from each of the different representations. Subsequently, we will focus on the selected model for each of the datasets and focus on the relevant themes and predictions from the models. In the next section, we will discuss parameter estimates and predictions that suggest that global similarity of the orthographic representations generally exerts a large influence on lures but does not substantially affect target items. After that, we will discuss the non-linear effect of similarity, with similarity effects being largest for high similarity items.

### Global similarity examples

**Fig. 5:**
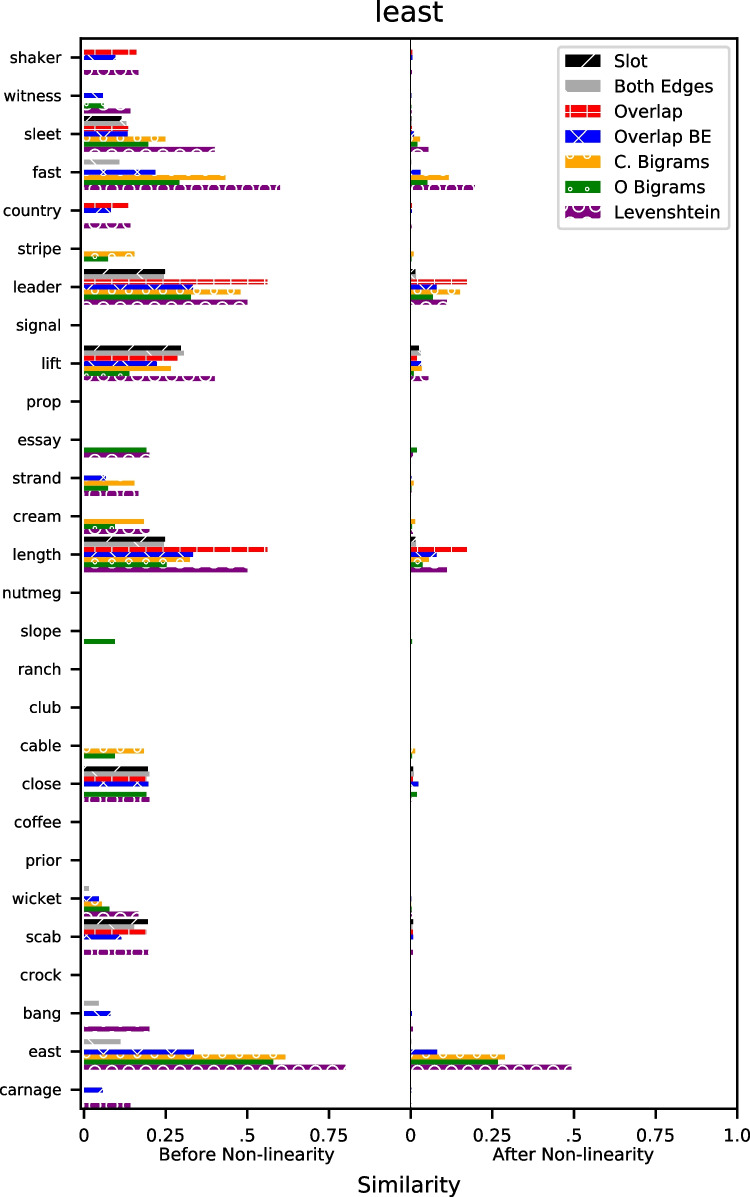
Illustration of global similarity from each of the representations from a trial in the Rae et al. dataset. The word at the top is the probe word, the words on the left are the study list words, and the *horizontal bars* are the similarities between the list words and the probe word before (*left*) and after (*right*) the non-linear transformation

Figure 5 shows an illustration of an example trial from each of the models from the Rae et al. dataset. Each of the words are words from the study list and the word at the top ("least") is the probe word for a particular trial. The horizontal bars represent the similarity from each of the underlying representations – the left and right columns show the similarity before and after the non-linear transformation of similarity using the estimated value of the power parameter *p* from the fits to data.

**Fig. 6:**
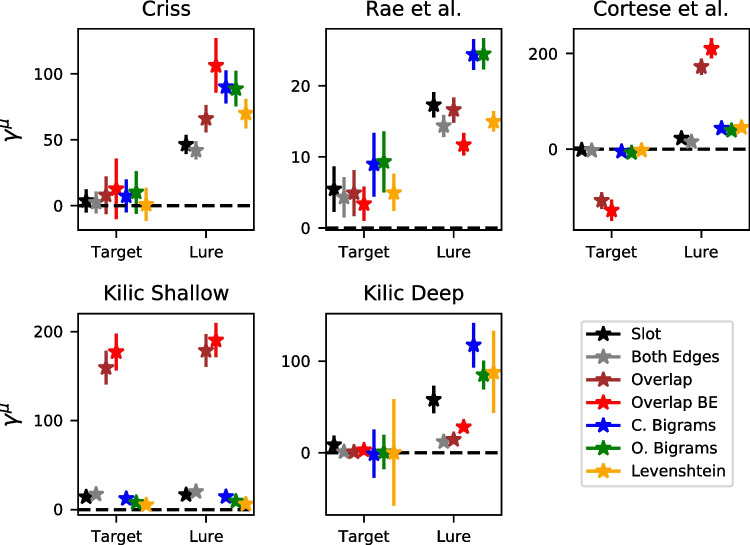
Group mean $\mu$ estimates of the scale parameter $\gamma$ for each model and dataset

Two trends are evident in the figure. First, the non-linearity reduces similarities and ensures that only items that range from moderate to high similarity "survive" the non-linear transformation. Essentially, this reduces the memory set to a smaller number of "effective" items. Second, there is generally agreement among the two classes of representations – the absolute position models (slots, both edges, overlap, and both-edges overlap models) produce comparable similarity values, as do the relative position models (closed and open bigrams). While the absolute and relative position models agree on some words, there are some words that they critically disagree on. For instance, the word "east" is on the study list – one can see that both the closed and open bigram models continue to describe it as a similar item after the non-linear transformation. The absolute position models instead all describe this as an item with essentially zero similarity, which is likely due to the fact that the omission of the first letter caused a misalignment between the studied word and the probe word that heavily penalized the resulting similarity.

We found this example trial by converting the similarities to z-scores and finding example trials that maximally discriminated between the models. In each of the models that favored relative position models, we found a number of similar trials where there were one or more list words where there was misalignment between those words and the probe words, such as the addition of a prefix.

### Global orthographic similarity affects lures more than targets: parameter estimates and model fits

Both the parameter estimates and the model predictions suggest that global orthographic similarity affects lures to a much greater extent than targets. Figure 6 shows the group mean $\mu$ estimates of the scale parameter $\gamma$, which governs the direction and extent to which global similarity enters the drift rate for an individual trial. An important caveat about the $\gamma$ parameter is its tradeoff with the power parameter *p* – as *p* increases, the inter-item similarities *s* decrease, which can be compensated by an increase in the scale parameter $\gamma$. For this reason, the $\gamma$ parameter is not comparable across datasets or models, as there is no guarantee that *p* will be equated across them. However, $\gamma$ is directly comparable across targets and lures because *p* is held constant across these item classes.

Figure 6 reveals that $\gamma$ is higher for lures than targets for virtually every dataset and model. The one exception is the shallow processing condition of the Kiliç et al. dataset, is a condition where participants were instructed to response to a prompt about the letters in each of the words from the study list. However, the same was not true for the deep processing condition from the same dataset, which involves an orienting task that was not focused on orthography. For each of the other datasets, $\gamma$ was close to zero for targets, implying that global similarity has no influence at all on the resulting hit rates or the accompanying latencies for target items.

The model predictions reveal a similar pattern. For each of the winning models in each dataset, we calculated the global similarity *g* for each trial across the entire posterior distribution. Subsequently, we averaged the global similarity values across the posterior distribution. We then divided the global similarity into a number of equal area bins where the number of bins depended on the size of the dataset. We used six bins for the Criss and Rae et al. datasets, eight bins for the comparably larger Cortese et al. dataset, and four bins for the shallow and deep processing conditions of the Kiliç et al. dataset. Within each of the bins, we computed the hit and false alarm rates along with the .1, .5, and .9 quantiles of the response time (RT) distribution for correct and error responses, which are the 10th, 50th, and 90th percentiles of the RT distribution, respectively. These summary statistics were computed for both the data and the model’s predictions.

**Fig. 7:**
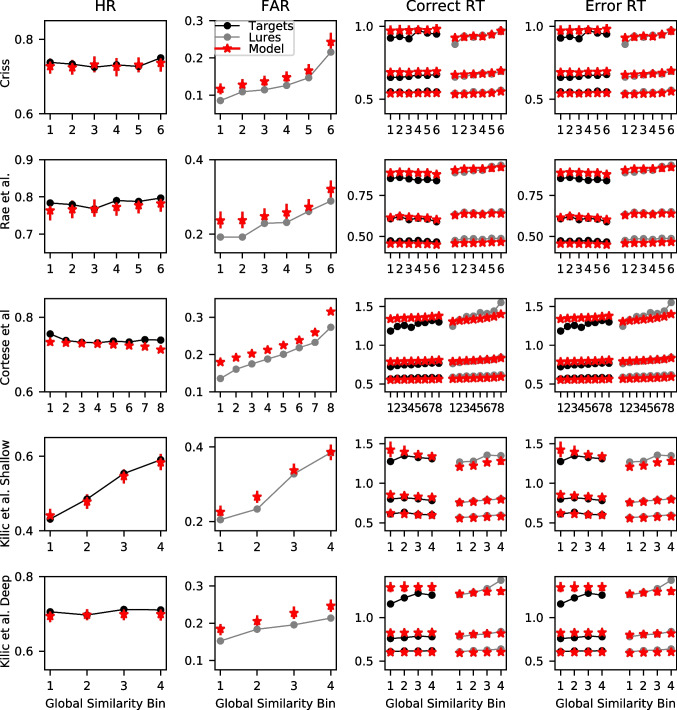
Group-averaged hit rates (HR: *first column*), false alarm rates (FAR: *second column*), correct RTs (*third column*), and error RTs (*fourth column*) for each of the global similarity bins (ranked from lowest to highest) from the data (*black*) and the winning models (*red*) of each dataset. RTs are summarized using the .1, .5, and .9 quantile. *Error bars* depict the 95% highest density interval (HDI)

These results can be seen in Fig. 7, which reflects the patterns seen in Fig. 6. We focused on the selected model for each dataset in part because space precludes depiction of all of the models, but also because each of the models were surprisingly similar in their predictions here. This is not to suggest that the models cannot be distinguished – as we discussed previously, their predictions differ substantially for some words. In the majority of the datasets, increases in global similarity – which are reflected in the higher bin numbers – result in substantial increases in the false alarm rate (FAR) along with slowing of the correct responses to lure items. The FAR increase from the lowest to the highest similarity bin is often .10 or higher. For context, this result parallels the FAR difference reported in the orthographic-phonemic condition of Shiffrin et al. (1995)’s Experiment 1, where they found an FAR of .096 for categories of two items and an FAR of .207 for categories of nine items (difference of  .10). The present results indicate that even lists of unrelated words can show substantial orthographic similarity effects that can be comparable to those from category length designs. The slowing of the RTs is most pronounced for the .9 quantile – the slowest RTs – whereas the fastest RTs (.1 quantile) and the median (.5 quantile) are relatively unaffected. This is broadly consistent with manipulations that selectively influence the drift rate (Ratcliff & McKoon, 2008) and also with our previous investigation focused on global semantic similarity (Osth et al., 2020).

It should also be mentioned that the FAR plots show some evidence of the non-linear similarity function that is due to the influence of the *p* parameter. Recall that increases in the power parameter *p* make it such that high similarity items tend to dominate the global similarity computation, meaning that there is relatively little difference between low and moderate similarity items but a comparably larger difference between high and moderate similarity items. This can be seen in the FARs for the Criss and Cortese et al. datasets, where the increase in FAR for the highest global similarity bin is larger than the other bins. We will return to evidence for non-linearity in the next sub-section.

Target items, in contrast, show little effect – the hit rates (HR) are fairly constant across each of the global similarity bins and there are minimal effects on target RTs. The one exception is the shallow processing condition of Kiliç et al., which shows a very large increase in the HR with increases in global similarity – a difference of .20 between the smallest and largest global similarity bin. However, the same result was not found in the deep processing condition, where there was little effect of global similarity on the HR and only a small effect of global similarity on the FAR. These results are consistent with the idea that depth of processing changes the nature of the underlying word representations. We will be returning to this issue later in the article when semantic representations are incorporated into the models.

Figure 7 also reveals that the winning models are providing an excellent fit to the data. A caveat is that there is somewhat of a circularity problem in evaluating the goodness of fit in this way – we have used the representations from the model to group the data, meaning that the differences between the bins that can be seen do depend on the model under consideration. This does not guarantee a perfect fit, however. One area where the models are often missing is that the RTs often change to a somewhat greater degree than the models predict. In the coming sub-section, we remedy the circularity problem by comparing the model’s predictions to an independent standard of similarity.

Global similarity exerting a larger influence on lures than targets is broadly consistent with a number of existing global matching models. In global matching models, the memory signal for lures is composed entirely of the global similarity, whereas for targets, it consists of the global similarity between the probe and the other list items and is additionally composed of the self-match, or the similarity between the probe and its own representation in memory. If the self-match is significantly larger than the inter-item similarity, then increases in the number of similar items will not exert a large influence on target items. In the REM model (Shiffrin & Steyvers, 1997) this occurs because the self-match distribution is extremely skewed. In the generalized context model (GCM: Nosofsky, 1991a) and the Minerva 2 (Hintzman, 1988) model, the non-linear similarity functions punish low similarity items. Since the self-match has high similarity by definition, the self-match dominates the global similarity for targets.

Figure 7 also reveals that the FAR are overpredicted in each of the datasets. A reviewer inquired as to whether this was a consequence of the orthographic representations exhibiting similarities to the probe items that are slightly excessive, or whether this was a consequence of the LBA architecture itself. In the Supplementary Materials, we report on fits to data of a simple LBA model with no orthographic representations. This model overpredicted the FAR to a similar degree, implying that this is a consequence of the LBA architecture itself.

### Lure similarity: the importance of non-linearity and exterior letters

#### Non-linear similarity functions enable the models to capture the difference in FAR between high and low similarity lures

In the previous sub-section, we depicted the model fits using the global similarity predictions from each of the models. To assess how well the models are performing in a way that circumvents the circularity problem, we used an independent standard from each of the selected models and classified each of the lures by their maximum similarity to the study list items. Specifically, we calculated the Levenshtein distance between the probe word and each of the studied words and selected the lowest distance as our measure of lure similarity. We defined words with Levenshtein distances of 1 (e.g., "dog" vs. "dogs"), 2 (e.g., "cargo" vs. "large"), 3 (e.g., "noodle" vs "nude"), or 4 or greater (e.g., "spider" vs. "shoe") as high, medium, low, and very low similarity, respectively. An alternative measure to the Levenshtein distance is the Damerau distance, which treats transpositions between adjacent letters as a single transformation (e.g., "trail" vs. "trial") while this would count as a distance of 2 according to the Levenshtein metric. However, we discovered in the process of analyzing these datasets that transposition pairs of words are quite rare in language and did not occur in any of our datasets, so there were no differences between the Levenshtein and Damerau distances.

In addition, as we previously mentioned, studies investigating priming effects in the lexical decision task have found evidence for the importance of the exterior letters of the words. To our knowledge, it has not been investigated in recognition memory as to whether exterior letters are more critical in orthographically similar lures. For this reason, in the high similarity lures, we investigated whether the missing letter was a start letter, an interior letter, or an end letter. Because high similarity lures were relatively rare in our datasets (ranging between 1 and 3% of all trials), the results are somewhat noisy for this comparison, but consistent enough across datasets to reveal a pattern.

For moderate and low similarity lures, we also investigated how the number of similar items on the list affects performance. That is, for each of these lure types, we looked at whether there were one (1x) or two (2x) items on the list with the same Levenshtein distance. We were unable to separate the data in this fashion for high similarity lures because of their rarity, and because the number of trials where there were two high similarity items on the study list was close to zero in many of our datasets.

**Fig. 8:**
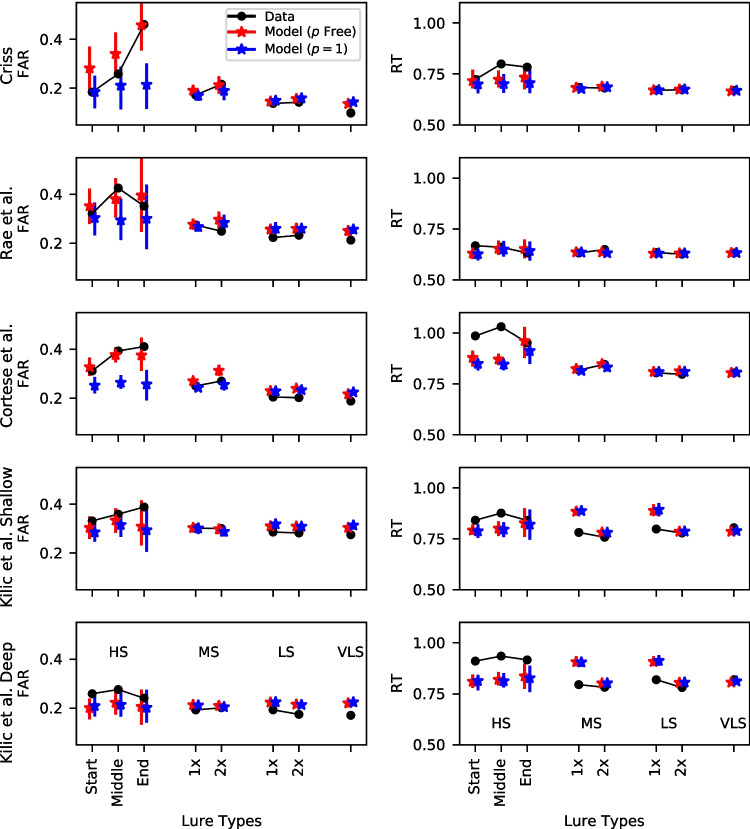
Group-averaged false alarm rates (FAR, *left column*) and median RTs (*right column*) to the lure types of varying degrees of similarity. The high similarity (HS) lures are sub-divided based on which letter in the word was missing (start = initial letter, middle = interior letter, end = terminal letter). *Error bars* depict the 95% highest density interval (HDI). Note: HS = high similarity, MS = medium similarity, LS = low similarity, VLS = very low similarity, 1x = one matching item on the study list, 2x = two matching items on the study list

Group-averaged false alarm rates (FAR) and median RT to lures from the winning models and the data for the lure categories described above can be seen in Fig. 8. Median RTs were depicted instead of the complete distribution as there were too few observations for high similarity lures. To illustrate the importance of non-linearity in capturing these trends, we additionally fit the same models to data with a linear similarity function ($p = 1$) – these models are depicted in blue. Note that we only fit linear versions of the winning models from Table 3 because fitting all of the models would result in a very large number of additional models. A model selection between the non-linear and linear models can be seen in Table 4, where it can be seen that the nonlinear models are selected decisively despite a larger number of parameters in three of the four datasets, with the exception of the Kiliç et al. dataset where the advantage of the nonlinear model is relatively small.

**Table 4:** $\Delta$ WAIC values for the models with a nonlinearity similarity function (*p* free) and the models with a linear similarity function ($p = 1$)

	Criss		Rae		Cortese		Kiliç	
Model	$\Delta$ WAIC	*P*	$\Delta$ WAIC	*P*	$\Delta$ WAIC	*P*	$\Delta$ WAIC	*P*
*p* Free	**0**	**21**	**0**	**23**	0	13	**0**	26
$p = 1$	145	20	51	22	371	12	2	24

See the text for details

What is apparent from Fig. 8 is the non-linear relationship between Levenshtein distance and the FAR – FAR are in some cases dramatically higher for high similarity (HS) than medium similarity (MS) lures, but the difference between medium similarity and low similarity (LS) is small, and the difference between low similarity and very low similarity lures (VLS) is barely noticeable. What is also interesting is that the difference between one and two medium similarity lures is fairly small – this difference is much smaller than the difference between medium and high similarity lures. While the models with non-linear similarity functions are able to capture these trends, the $p = 1$ models with linear similarity functions are unable to capture the large difference in FAR between high and medium similarity lures in three of the four datasets. An exception is the Kiliç et al. dataset, where the difference between the linear and non-linear models is quite small, which explains why the linear model was favored slightly in the model selection.

Several of the models made very similar predictions for each of the datasets. One exception was for the Levenshtein model, which generally accounted for the data well but was unable to capture the differences between the high similarity (HS) lure types given that the model does not differentially weight transformations based on letter position. However, as it turns out, the Cortese et al. (2015) dataset revealed very different predictions for the absolute and relative position models. Specifically, all of the absolute position models were unable to capture the larger FAR for the HS lures, while both the closed and open bigram models were able to capture this difference. Figures comparing these models can be seen in the Supplementary Materials.

**Fig. 9:**
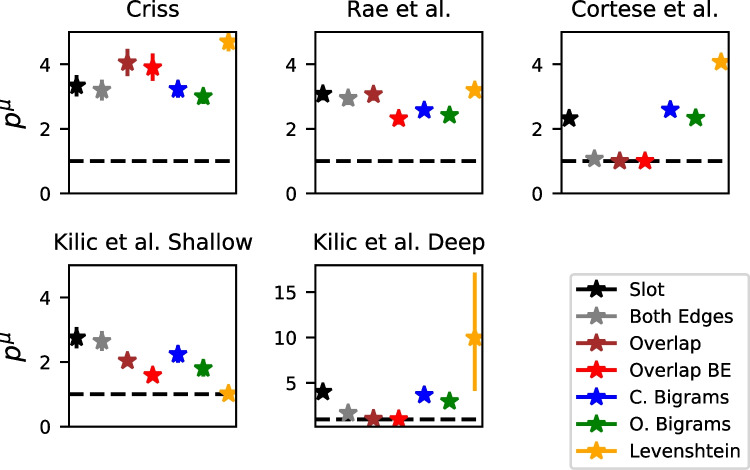
Group mean $\mu$ estimates of the nonlinearity parameter *p* for each model and dataset

Estimates of the group mean $\mu$ of the *p* parameter for each model and dataset can be seen in Fig. 9. One can see that the estimates differ considerably across both datasets and models. For the Criss and Rae et al. datasets, the estimates hover around 3.0, which is the nonlinearity parameter in the Minerva 2 model (Hintzman, 1988). For the Kiliç et al. dataset, the overlap model – which was the selected model – was close to linear ($p = 1$) in the deep processing condition, which may explain why its predictions were similar to the linear model. The overlap models and the both-edges slot code model all received estimates of *p* that were close to 1 in the Cortese et al. dataset, which may explain why the models were unable to capture the differences between high and low similarity lures depicted in the Supplementary Materials.

#### The importance of the initial letter

Another trend in Fig. 8 is the importance of the initial letter in each word. In the Criss and Cortese et al. datasets in particular, there were lower FAR to high similarity lures that lacked the initial letter than to lures that lacked an interior or end letter. This tendency was present in the Rae et al. dataset, although it was somewhat noisier. The effect was not noticeable in the Kiliç et al. dataset, but interpretation of this dataset is complicated by the fact that the encoding tasks in the depth of processing manipulation may have guided attention away from the exterior letters during the study phase.

**Fig. 10:**
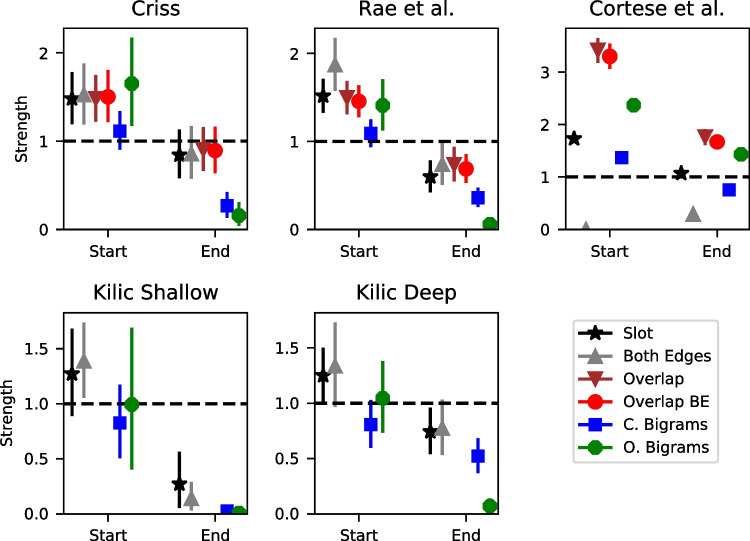
Group mean $\mu$ estimates of the initial letter strength $\alpha$ and terminal letter strength $\Omega$ for each model and dataset. *Error bars* indicate the 95% highest density interval (HDI)

A surprising result was that high similarity lures that mismatched the final letter exhibited *larger* FAR in some cases. This tendency was most pronounced in the Criss dataset but was also a small trend in the Cortese et al. dataset and the shallow processing condition of the Kiliç et al. dataset. Both trends are corroborated by an analysis of the parameter estimates of the strengths of the exterior letters. Figure 10 shows estimates of the posterior distribution of the group mean of the start letter strength $\alpha ^\mu$ as well as the end letter strength $\Omega ^\mu$ for each of the models. The dashed lines in the figure indicate the strength of the interior letters (1). For each model class, it is clear that the start letter is represented with more strength than the end letter in almost every dataset, suggesting a within-word primacy effect but not a recency effect.

A surprising result was that there were several cases where the end letter was represented with less strength than the middle letters, namely for the Criss, Rae et al., and Kiliç et al. datasets. There was some disagreement between the models in the estimates of $\Omega ^\mu$ that is likely due to the differences in the way the end letter was treated. In bigram models, lower similarity is produced anytime there is a difference in the end letter between the probe and studied strings. The absolute position models are complicated by the fact that differences in word length between the probe and studied strings can result in mis-alignment of the exterior letters.

## Integration of semantic representations

Up to this point, we have established that orthographic representations can be quite consequential in determining memory performance. Specifically, false alarm rates (FAR) increase to a large extent with the increase in the global similarity of orthographic representations and FAR are much higher to highly similar orthographic lures than to medium or low similarity lures, even though the datasets were constructed from lists of unrelated words without any obvious categorical structure. While such results are contrary to the idea that long-term memory primarily consists of semantic representations (e.g., Baddeley, 1966), the obvious question remains – how consequential are orthographic representations compared to semantic representations?

To address this question, we constructed new global similarity models that additionally incorporate the similarity of semantic representations. We constructed our semantic similarity measures from representations from the word2vec model (Mikolov et al., 2013), a neural network model of word embeddings. Word2vec has had success in outperforming other models of semantic representations in capturing priming data in the lexical decision task (Mandera et al., 2017) and additionally has been successful in capturing false memory phenomena in the Deese-Roediger-McDermott (DRM) paradigm (Gatti et al., 2022). We used the pre-trained vectors from the FastText library (Grave et al., 2018) that were trained on a complete Wikipedia corpus using word information only. We defined the semantic similarity *c* between a probe *i* and a studied item *j* as:

14 $$\begin{aligned} c_{ij} = \text {max}(\text {cosine}_{ij}, 0) \end{aligned}$$

where the cosine indicates the cosine of the angle between the two vectors. The cosine similarity metric is similar to the dot product, but is normalized for vector length and bounded on a [-1,1] interval. We truncated the cosine values at zero for two reasons. First, a cosine value of zero occurs when vectors are orthogonal to each other, which indicates maximum dissimilarity, making values below zero hard to interpret. Second, applying the power transformation to similarities reduces those values even further – by truncating the similarity values to zero, the power transformation can only reduce values to zero.

The global similarity *g* for a probe *i* is now:

15 $$\begin{aligned} g_i = \sum \limits _{j \in L, j \ne i}{(w_os^p_{ij} + (1 - w_o)c^{p*}_{ij})} / N_L \end{aligned}$$

where $w^o$ is the weight of orthographic information and $p*$ is the power parameter for semantic similarities. The weight parameter $w^o$ is analogous to selective attention weights in the generalized context model (Nosofsky, 1986; Nosofsky et al., 2011; Osth et al., 2023) and reflects the fact that participants may prioritize one dimension over the other in their encoding of the items during either the learning or the test phases. We applied a separate power parameter for semantic similarity as the two types of similarity may occur on different scales.

For each dataset, we used the selected orthographic representation from Table 3, namely the closed bigrams for the Criss dataset, the open bigrams for the Cortese et al. and Rae et al. datasets, and the overlap model for the Kiliç et al. dataset. The models were applied to data in the same manner as the models earlier in the article. For the Kiliç et al. dataset, we allowed parameters corresponding to semantic encoding to vary with the depth of processing conditions, namely the $w_o$ and $p*$ parameters.

### Orthographic similarity is as consequential as semantic similarity

**Fig. 11:**
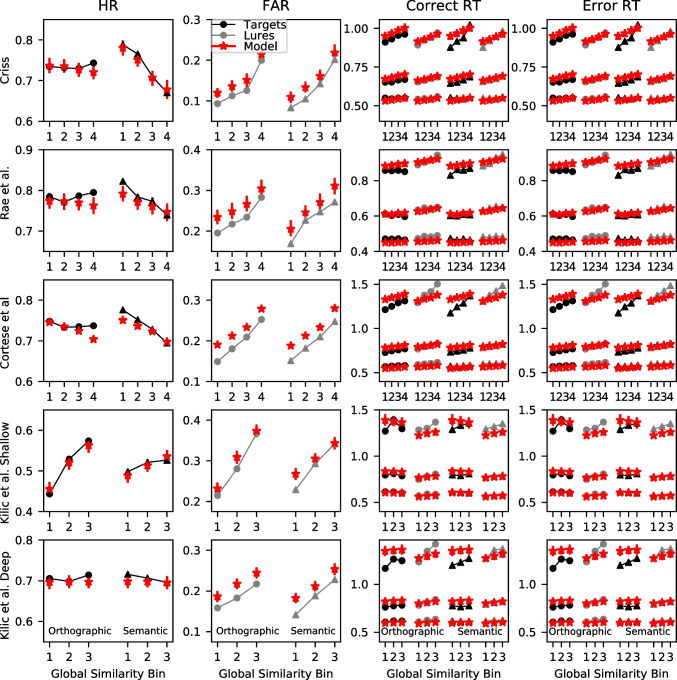
Group-averaged hit rates (HR: *first column*), false alarm rates (FAR: *second column*), correct RTs (*third column*), and error RTs (*fourth column*) for each of the orthographic and semantic global similarity bins (ranked from lowest to highest) from the data (*black*) and the winning models (*red*) of each dataset. RTs are summarized using the .1, .5, and .9 quantile. *Error bars* depict the 95% highest density interval (HDI)

To understand the relative influences of orthographic and semantic similarity, for each probe word, we separately calculated the global similarity of the semantic and orthographic representations in isolation. For each type of global similarity, we then divided the trials into a number of equal area bins, specifically three bins for each dataset with the exception of the Cortese et al. dataset, where we used four bins due to the comparably larger size of the dataset. For each global similarity bin, we then calculated the group-averaged hit rate, false alarm rate, and RT quantiles for correct and error responses – these results can be seen in Fig. 11.

What is apparent from the figure is that for the three datasets that lack a depth of processing manipulation (the Criss, Rae et al., and Cortese et al. datasets), there is a striking resemblance in the false alarm rates (FAR) for both orthographic and semantic similarity – there are sizeable increases in the FAR that are nearly equivalent in magnitude. They are also accompanied by slowing of the response times (RTs) that is most pronounced for the slowest RTs (the .9 quantile). The key difference between the effects of orthographic and semantic similarity appear to rest in the hit rates (HR) – HRs decrease with increasing semantic similarity for these datasets, while they appear to be relatively constant with increasing orthographic similarity. However, it is also important to note that semantic similarity is confounded with word frequency (Osth et al., 2020), and thus it is very likely that the higher semantic similarity bins contain a higher proportion of words of higher natural language frequency, which often exhibit lower hit rates (Glanzer & Adams, 1985).

Results for the Kiliç et al. dataset depend on the depth of processing condition. The shallow processing condition shows a larger effect of orthographic than semantic similarity, in which the increase in the HR and FAR is larger for increasing orthographic similarity than semantic similarity. The deep processing condition shows the opposite for the FAR in that the increase in the FAR is larger for semantically similar items than for orthographically similar items, although the trend is weaker overall. This may be due to the fact that FAR are reduced in the deep processing condition relative to the shallow processing condition. In addition, the hit rate did not noticeably change with increases in either similarity type.

**Fig. 12:**
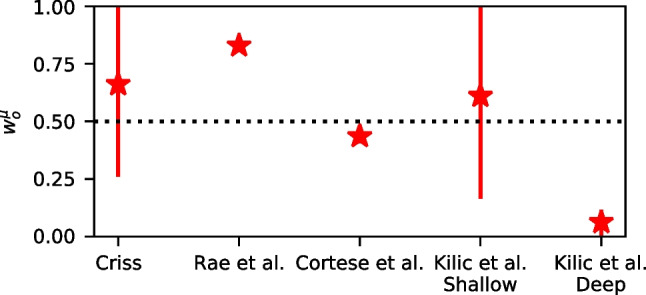
Group mean $\mu$ estimates of the weight on orthographic similarity $w_o$ for each dataset. *Error bars* indicate the 95% highest density interval (HDI)

These results are also reflected in the estimates of the weight on orthographic similarities $w_o$. Figure 12 shows estimates of the group mean $w_o^\mu$ for each dataset. High uncertainty was observed in both the Criss dataset and the shallow processing condition of the Kiliç et al. dataset which was due to a high degree of variability across participants – this was confirmed in analyses of individual participant parameters and group-level standard deviation parameters in the Supplementary Materials. What is noteworthy otherwise is that only one case indicates a dominance of semantic similarity, namely the deep processing condition of the Kiliç et al. dataset. Otherwise, each dataset indicates that the weight of orthographic similarity is comparable to that of semantic similarity, and in at least one case (the data of Rae et al.) shows a dominance of orthographic similarity.

The fact that the different depth of processing conditions changes the emphasis on orthographic versus semantic similarity is consistent with previous findings that participants’ false recognition of different lure types depends on the nature of the encoding task. Specifically, it has generally been found that false recognition of semantic lures is higher when an encoding task emphasizes semantic processing while false recognition of perceptually similar lures is prevalent when the encoding task emphasizes the processing of the perceptual characteristics of the words (Chan et al., 2005; Elias & Perfetti, 1973; Jacoby, 1973).

An important caveat on the estimates of $w_o^\mu$ in Fig. 12 is that they will critically depend on the ability of each representation to capture the data. A deficiency in one representation will naturally produce a stronger weight on the other representation, as the deficient representation will not be able to capture much variation across items. We have tried to remedy this problem by only using the best of the tested orthographic representations present in Fig. 3. While we did not perform model selection, word2vec is considered the state-of-the-art semantic representation and has outperformed many other models such as latent semantic analysis (Mandera et al., 2017).

### Capturing performance for individual items

One of the unique advantages of supplying representations for individual items in a global similarity framework is the ability to make predictions for individual items in an experiment. As mentioned previously, a unique strength of the Cortese et al. (2015) dataset is that performance for individual items can be assessed, mainly because there was a large number of participants and each participant was tested on the same set of items. The underlying logic is that items that are more similar to other items in the memory set are more likely to receive higher hit and false alarm rates than items that are dissimilar to other items. While this dataset randomized items across study lists such that each word is likely to be accompanied by different words on each study list, words that are more similar to other items in the larger set of words are more likely to be accompanied by similar items on each study list.

**Fig. 13:**
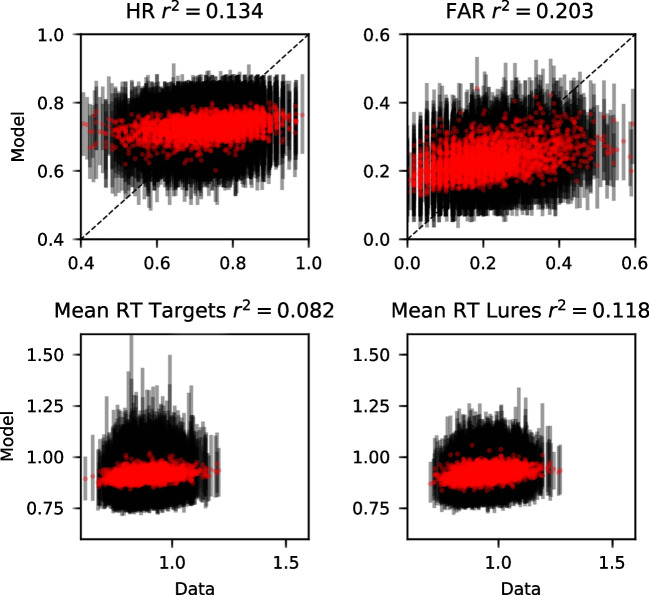
Scatterplots depicting the hit and false alarm rates along with the mean RT for targets and lures for the 3000 words from the Cortese et al. dataset. The *red dots* indicate the mean of the posterior predictive distribution while the *black lines* indicate the 95% highest density interval (HDI) of the posterior predictive distribution. See the text for details

For this dataset, we used the combined orthographic and semantic global similarity model and generated the posterior predictive hit rate, false alarm rate, and median RT for targets and lures, and contrasted these with the data. These results can be seen in Fig. 13, where the black lines indicate the 95% highest density interval (HDI) of the model predictions, and the $r^2$ estimates were calculated from the mean of the posterior predictive distribution using Spearman’s correlation’. At first glance, the $r^2$ estimates are not encouraging. However, there are two important considerations. First, the uncertainty in the predictions is visibly very large, which imposes limitations on the maximum $r^2$ value that can be obtained. This is in part due to the fact that with close to 120 participants, each item received roughly 60 target and lure trials each, which is not extensive. In addition, the model predictions for each item vary across participants depending on the accompanying words on each presented study list.

Second, we would like to note that the $r^2$ estimate for the FAR ($r^2 = .209$) is higher than the $r^2$ estimate from the original authors’ multiple regression with six predictors on the same dataset. Specifically, Cortese et al. (2015) regressed age of acquisition, word frequency, imageability, word length, along with both orthographic and phonological density measures and obtained an $r^2$ of .147. Our model, in contrast, exceeded the performance of their model in predicting FAR with a single global similarity measure that is comprised of orthographic and semantic representations. This is impressive when one considers that our model is constrained not just by the variability across the items, but also the variability across participants and the simultaneous constraint of the RTs.

Nonetheless, the Cortese et al. (2015) regression model considerably outperforms our model in its predictions for hit rates, where it received a performance of $r^2 = .350$. Not only does our model perform considerably worse than this, but Fig. 13 reveals that the predictions for hit rates are considerably narrower in range than the data.

**Table 5:** The ten words with the highest hit rate (HR) and false alarm rate (FAR) from Cortese et al. (2015) dataset along with the predictions of the global similarity model that contains both orthographic and semantic representations. See the text for details

	HR		FAR	
Rank	Data	Model	Data	Model
1	brothel	abbot	converse	complete
2	orgy	kazoo	convey	create
3	whoopee	vigil	banquet	protect
4	buckle	yahoo	convert	restrain
5	voodoo	jaguar	decent	certain
6	condom	ruckus	success	nature
7	virgin	gallows	content	sterile
8	humbug	voodoo	descent	retain
9	paisley	syrup	rapid	relate
10	limbo	gangrene	defense	desire

Why does this occur? Table 5 shows the ten words with the highest hit and false alarm rates from both the data and the model. Inspection of the words with the highest HR words in the data reveal that many of these words may be of particular interest to undergraduate students, which partly explains the deflections of the model from the data.

However, a more critical theoretical limitation of the present work is that our modeling omitted self-similarity in predictions for target items, such that the global similarity measure is purely comprised of inter-item similarities. This implies that target items that the only factor that makes a target item receive a high hit rate is its dissimilarity to other items in the memory set. Consequently, the model’s predictions for a given item’s hit rate is perfectly correlated with its predicted false alarm rate. The data instead show a very weak negative correlation ($r = -.046$, $p = .011$), indicating that some variable other than inter-item similarity is contributing to variation in the hit rates.

While a natural recourse would be to include the self-similarity measure, it is important to note that for both the orthographic representation (open bigrams) and for the semantic similarity measure (vector cosine) the self-similarity always has the same value – one. Because this measure does not vary across trials, it will not meaningfully assist in capturing the variation in the hit rates. In the General discussion, we note other sources of variability that could be included to improve the fit of the model.

## General discussion

The present work aimed to explore the global similarity predictions with orthographic representations. Specifically, we explored a range of different representations from the psycholinguistics literature, including models where words are represented in terms of the absolute positions of the letters – slot codes and overlap models – along with models where the words are represented in terms of the relative positions of the letters – bigram and open bigram models.

Each representation was explored as a separate global similarity model that determines drift rates for individual trials in a linear ballistic accumulator (LBA: Brown & Heathcote, 2008) framework. The models were fit to four different recognition memory datasets where the study lists were comprised of unrelated words. While a single representation was not favored consistently across all of the datasets, three of the datasets preferred relative position models while one of the datasets (Kiliç et al., 2017) preferred a model where letters are represented in terms of the absolute position. A key difference with this dataset is that words were encoded with two different encoding tasks – a shallow processing task where participants had to identify whether an "e" was present in the word or a deep processing task where participants had to rate the pleasantness of a word. It is possible that these encoding tasks may have changed the way participants represented letter order. In fact, the results demonstrated that word representations in the shallow processing task represented the letter "e" more strongly and several of the parameters governing the orthographic representations changed across the two conditions. Nonetheless, the dataset of Cortese et al. (2015) – which was the largest dataset – was very diagnostic of the different models and only the relative position models were able to provide a good account of these data (see the Supplementary Materials for more information).

For each dataset, the preferred global similarity model demonstrated that the false alarm rate (FAR) increased to a fairly sizeable extent with increases in the global similarity of the orthographic representations. When semantic representations were included in the model, increases in global orthographic similarity produced comparable effects on the FAR as increases in global semantic similarity. Parameter estimates of the weight of each representation produced a similar interpretation – orthographic representations often yielded weights that were comparable to those of semantic representations and in some cases even exceeded them. These results are contrary to the claims that long-term memory is comprised mainly of a semantic code (Baddeley, 1966).

Another result of the modeling is that parameter estimates revealed a clear importance of the initial letter in determining similarity between letter strings. This was additionally reflected in the data, where it was found that high similarity lures that did not contain the start letter exhibited lower false alarm rates compared to lures that did not contain an interior or terminal letter, which can be considered a within-word primacy effect. To our knowledge, there have been no studies investigating this question in the recognition memory literature, despite a fair number of studies investigating the relative importance of exterior and interior letters in the reading literature (Grainger & Jacobs, 1993; Jordan et al., 2003; Scaltritti et al., 2018; Whitney, 2001). One crucial difference in our results is that we did not find any special importance of the terminal letter, with some datasets showing *weaker* importance of the terminal letter compared to interior letters. While most studies in the reading literature have similarly found a special importance of the terminal letter, some studies have only found advantages for the initial letter (Whitney, 2001).

### Adjudicating between the orthographic representations

As mentioned above, the model selection in the present work did not clearly adjudicate between each of the orthographic representations. This begs the question – which additional constraints can be imposed that can better select between them? One possibility would be to test lure words that are transpositions of studied words. Transposition strings can be constructed by swapping adjacent letter pairs, such as the difference between "trial" and "trail." In the psycholinguistics literature, a major constraint on orthographic representations is that transposition strings are more effective primes than non-transposed strings that are either one or two letters different from the target word (Andrews, 1996; Perea & Lupker, 2003a, b), implying that pairs of transposition strings are more similar to each other than the other pair types.

The transposition effect imposed strong constraints on several of the models we considered here. For instance, the slot code model cannot produce transposition effects because the swap results in the two letters being out of alignment with each other ("trail" and "trial" have 3/5 matches with each other), which predicts that transposition strings should be just as effective as any other prime that is two letters apart from the target word. A similar problem arises with closed bigrams, wherein the transposition results in the loss of two bigram matches. Transposition advantages can instead be captured by both the overlap and open bigram models. The overlap model captures the effect because the uncertainty in the representation of letter position means that even if letters are out of alignment, they can still partially match the target word. The open bigram model can capture the effect because the long-range bigrams are still preserved across the transposition prime and the target string.

A promising approach to testing between the representations would be to evaluate whether transposition lures produce higher false alarm rates than lures that are one or two letters different from a target word. We were not able to test for this possibility in the present work because none of the datasets contained pairs of words that were transpositions of each other. In work conducted after the work in the present manuscript, we ran experiments testing for this possibility in recognition memory by explicitly controlling lure similarity and indeed confirmed that transposition lures exhibit higher false alarm rates than lures that are one or two letters different from a target word (Zhang & Osth, submitted). While both the overlap and open bigram models were capable of addressing the effect, only the open bigram model was able to capture the size of the effect. Subsequent analyses revealed that the estimated uncertainty parameters in the overlap model were too small to predict large transposition effects.

### Implications for episodic memory models

An increasingly popular trend in models of episodic memory is to incorporate realistic semantic representations to capture similarity effects or false memory intrusions (Johns et al., 2012; Kimball et al., 2007; Morton & Polyn, 2016; Osth et al., 2020; Polyn et al., 2009; Reid & Jamieson, 2023). The present work demonstrates that such models could similarly benefit from the inclusion of orthographic representations. As mentioned previously, when orthographic or phonemic categories are constructed in the Deese-Roediger-McDermott (DRM) paradigm (Roediger & McDermott, 1995), very high false alarm rates are found to the critical lure that are found that are often comparable in magnitude to the effects seen in semantic DRM studies (Chang & Brainerd, 2021; Coane et al., 2021; Sommers & Lewis, 1999; Watson et al., 2003). While several of these models have been able to address semantic DRM effects, orthographic representations may enable such models to simultaneously address perceptually-driven DRM effects. The only model we are aware of that includes orthographic representations is the REM model presented by Steyvers (2000), which uses a slot code representation. However, the present work demonstrated that the slot code was outperformed by other representations in virtually every dataset, suggesting that other models may benefit from the inclusion of bigram or open bigram representations.

Several of these models, however, implement word representations using vectors and represent the similarity between vectors using dot products or cosines between the vectors, whereas the present work has instead used a feature matching scheme. How then could the orthographic and semantic representations be integrated together? Previous work has demonstrated that it is possible to construct vector representations of each of the orthographic representations in this manuscript (Cox et al., 2011; Hannagan & Grainger, 2012). For instance, Cox et al. (2011) were able to construct orthographic representations using holographic representations, similar to those from BEAGLE (Jones & Mewhort, 2007) and the TODAM model (Murdock, 1982). Specifically, each letter was represented as a vector with a finite number of elements where each element was sampled from a normal distribution. Bigram representations can be constructed by binding letter representations together using circular convolution, which is how the TODAM model represents bindings between words or items on a study list (Lewandowsky & Murdock, 1989; Murdock, 1982). Slot codes were constructed by binding letters to vectors corresponding to the position of the letter in the word. While Cox et al. (2011) did not implement a holographic vector implementation of the overlap model, an analog of the model could be constructed by representing each word in every position and weighting each of the letters using a Gaussian function. In a memory model, such orthographic vectors can be integrated with semantic vectors either by concatenating the two vectors together into a single large vector, or alternatively vector dimensionality could be preserved by binding the two vectors together using circular convolution.

### Improving the ability to capture individual items

In addition to capturing more general orthographic and similarity effects above, another advantage of incorporating stimulus-specific representations into global similarity models is that it enables the models to make predictions about individual items. Existing approaches to understanding word memorability have focused on exploring how variations in the properties of the words themselves, such as word frequency, animacy, neighborhood size, and aspects of the semantic representations relate to variations in word memorability (Aka et al., 2023; Cortese et al., 2010, 2015; Cox et al., 2018; Madan, 2021). While these properties have been able to account for significant proportions of variation in the memorability of the items, there is a sense in which approaches like this "kick the can further down the road" because it invites further questions – why are these properties able to explain memorability across stimuli? Without a formal theory, there may not be clear answers to these questions.

An advantage of the global similarity framework is that it provides clear answers to these questions. An item will be memorable to the extent to which it matches its own representation (if it is a target item) and the other items in the memory set. Global similarity models have enjoyed a great deal of success in explaining variations in item memorability with simple, low-dimensional nonword stimuli (e.g., Busey & Tunnicliff, 1999; Nosofsky, 1991b; Nosofsky et al., 2011). When we applied our model to the Cortese et al. (2015) data, however, while we achieved a modest ability to capture variations in the false alarm rate, the model’s account of the item variation in hit rates was lacking. A possible limitation there is that our global similarity measures considered only the inter-item similarity. While we could incorporate self-similarity, the majority of our representations and similarity metrics would produce self-similarity values of one that do not vary across items.

Nosofsky and Meagher (2022) noted a similar problem in their modeling of item variation in recognition memory for naturalistic images of rocks, in which their global similarity model, namely the generalized context model (GCM), achieved a reasonable $r^2$ for the false alarm rates but was almost completely unable to capture any variation in the hit rates. Their model similarly assumed that all self-similarities have a match value of one. However, they were able to greatly improve the model’s ability to capture the hit rates when the self-similarity was augmented with distinctive features from a feature rating study. Specifically, they incorporated a hybrid similarity model, where similarity was driven by continuous measures – an exponential transformation of distance between items in a multidimensional space – along with discrete features derived from the feature rating study (Knapp et al., 2006; Nosfosky & Zaki, 2003). The combination of these features not only allowed the self-similarities to exceed one, but they varied across items as a function of the number of features rated for each stimulus.

While a similar approach could in principle be applied here, there is a question as to what features would be considered distinctive features in individual words. One potentially fruitful avenue would be to consider the rarity of orthographic features in natural language. In the present work, all orthographic features – whether they were letters in position or bigram matches – were equally weighted in the similarity calculation.

However, in recognition memory, there is evidence that rare features may be more consequential than common features. Specifically, Malmberg et al. (2002) manipulated word frequency and letter frequency in a 2x2 design and found that words with rare letters exhibited slightly higher hit rates and considerably lower false alarm rates than words with common letters. Thus, it is possible that words with higher hit rates contain features that are weighted more heavily due to their rarity in language. Nonetheless, future work on letter frequency effects is required to ascertain whether they are required to be incorporated into orthographic representations. Another possible interpretation of the Malmberg et al. (2002) results is that words with rare letters exhibited fewer false alarm rates simply because such words were less likely to overlap with other words on the study list, and may not reflect different weights of the letters per se.

Another finding that suggests that feature weight should vary across items comes from Mewhort and Johns (2005). In their experiments, rejections advantages were found for words containing letters exterior letters that were not present on the study list even if the other exterior letter matched multiple items on the study list. Specifically, they compared lures containing one exterior letter that was not present on the study list (the extralist feature) along with another exterior letter that matched two items (2:0 lures) to words where both exterior letters matched one study list item (1:1 lures). 2:0 lures exhibited lower FAR and faster RTs, suggesting that the extralist feature was more consequential than the strongly matching feature.

The extralist feature effect is similar to the feature frequency effect in that it suggests that features should be differentially weighted. However, where the two effects are critically different is that the feature frequency effect suggests a sensitivity to the long-running frequencies in language whereas the extralist feature effect suggests a sensitivity to the letters that were present on a specific and recent episode (the study list). Recently, Osth et al. (2023) accounted for the extralist feature effect using a model where attention to the features or stimulus values is inversely proportional to how well represented they were on the study list. Although they only accounted for visual non-word stimuli, such an account could potentially apply to orthographic representations as well, which may improve a global similarity model’s ability to capture variation in false alarm rates across items. However, to our knowledge, there has yet to be a unifying explanation of the extralist feature effect and the feature frequency effect.

A further limitation is that we have assumed in the present work that only the study list items contribute to the global similarity computation. In other words, only *item-noise* contributes to performance. In practice, items from previous lists or pre-experimental contexts can additionally contribute to performance, such as prior occurrences of the cue – referred to as *context-noise* (Anderson & Bower, 1972; Dennis & Humphreys, 2001; Reder et al., 2000), – or prior occurrences of items that mismatch the cue – referred to as *background noise* (Murdock & Kahana, 1993). Each source of interference can in principle be integrated into a single model (e.g., Fox et al., 2020; Fox & Osth, in press; Osth & Dennis, 2015; Osth et al., 2018; Yim, Osth, Sloutsky, & Dennis, 2022). However, a challenge when incorporating realistic stimulus representations is assessing the participant’s history of each word prior to the experiment. One promising avenue is to quantify participant’s experiences with words using e-mails or their digital language histories (Yim et al., 2020), which would enable item-by-item estimates of pre-experimental interference. This could also serve to partly account for the word frequency effect in a manner that is independent of semantic similarity.

## Open practices statement

The data and model code for all of the work in this manuscript is available in our OSF repository (https://osf.io/hyt67/.). None of the modeling was preregistered.

### Supplementary Information

Below is the link to the electronic supplementary material.

Supplementary file 1 (pdf 263 KB)

## Appendix

### Additional details of the fitting procedure

#### LBA parameterizations for each dataset

##### Criss (2010) Experiment 2

Since strength was manipulated across lists, the *B* parameter was allowed to vary across the strength conditions, resulting in four *B* parameters ($B_{\text {old,strong}}$, $B_{\text {old,weak}}$, $B_{\text {new,strong}}$ $B_{\text {new,weak}}$.) The drift rate *V* was allowed to vary across word frequency (low frequency, or LF and high frequency, or HF words), targets and lures, and list strength conditions, resulting in a total of 8 $V_0$ parameters. Only a single value of *A*, $t_0$, $\eta _{\text {target}}$, and $V_0$ were estimated. In total, there were 16 base LBA parameters common to all models.

##### Rae et al. (2014)

Following Rae et al. (2014) and other subsequent investigations (e.g., Osth et al. 2017), we allowed several parameters to vary across the speed–accuracy emphasis conditions, including *A*, $t_0$, and *V*. Drift rate was additionally allowed to vary across word frequency (LF and HF words), resulting in a total of 8 *V* parameters. Only a single value of $\eta _{\text {target}}$ and $V_0$ were estimated. In total, there were 18 base LBA parameters common to all models.

##### Cortese et al. (2015)

Thresholds varied across old and new decisions ($B_{\text {old}}$ and $B_{\text {new}}$) and drift rate *V* varied across targets and lures ($V_{\text {target}}$ and $V_{\text {lure}}$). Only a single value of $t_0$, *A*, $V_0$, and $\eta _{\text {target}}$ were estimated. In total, there were eight base LBA parameters common to all models.

##### Kilic et al. (2017)

Since depth of processing was manipulated across lists, the *B* parameter was allowed to vary across the depth of processing conditions, resulting in four *B* parameters ($B_{\text {old,strong}}$, $B_{\text {old,weak}}$, $B_{\text {new,strong}}$ $B_{\text {new,weak}}$). The drift rate *V* was allowed to vary across both the depth of processing conditions and targets and lures, resulting in four parameters ($V_{\text {old,strong}}$, $V_{\text {old,weak}}$, $V_{\text {new,strong}}$, and $V_{\text {new,weak}}$). Only a single value of *A*, $V_0$, and $\eta _{\text {target}}$ were estimated. In total, there were 12 base LBA parameters common to all models.

### Data exclusions

For each dataset, we removed trials with response times (RTs) that were either very fast or slow, as these are likely to indicate guessing or random responding. For both the Rae et al. and the Criss datasets, we removed trials with RTs shorter than .2 s and slower than 2.5 s, resulting in the exclusion of less than 1% of the data. For the Cortese et al. dataset, we removed trials with RTs shorter than .2 s and slower than 4 s, which resulted in the exclusion of 2.5% of the data. In addition, one participant (#124) was excluded from the data as the datafile indicated that some of the study list lengths did not match the prescribed list lengths.

For the Kiliç et al. dataset, three participants were excluded for poor performance or a high proportion of fast RTs. One participant (#1) was excluded for exhibiting chance-level performance ($d' = -.001$). Two participants (#27 and #33) were excluded for exhibiting for a high proportion of fast guessing – 63.3% and 30% of RTs were below .3 s and both participants exhibited close to chance-level performance ($d' ~.1$). After these exclusions, trials with RTs faster than .3 s or slower than 3 s were excluded - this resulted in the exclusion of 3% of the data.

### Prior distributions on model parameters

Many of the individual parameters are sampled from group-level distributions with a defined mean $\mu$ and standard deviation $\sigma$. Some of the parameters which have a lower bound of zero were sampled on a log scale to improve sampling.

$$\begin{aligned} t_0\sim & {} \text {Normal}_{(0,\inf )}(t_0^\mu , t_0^\sigma ) \nonumber \\ A\sim & {} \text {Normal}_{(0,\inf )}(A^\mu , A^\sigma ) \nonumber \\ B\sim & {} \text {Normal}_{(0,\inf )}(B^\mu , B^\sigma ) \nonumber \\ V_0\sim & {} \text {Normal}_{(0,\inf )}(V_0^\mu , V_0^\sigma ) \nonumber \\ V_{\text {target}}\sim & {} \text {Normal}_{(0,\inf )}(V_{\text {target}}^\mu , V_{\text {target}}^\sigma ) \nonumber \\ V_{\text {lure}}\sim & {} \text {Normal}_{(0,\inf )}(V_{\text {lure}}^\mu , V_{\text {lure}}^\sigma ) \nonumber \\ \eta _{\text {target}}\sim & {} \text {Normal}_{(0,\inf )}(\eta _{\text {target}}^\mu , \eta _{\text {lure}}^\sigma ) \nonumber \\ \gamma\sim & {} \text {Normal}(\gamma ^\mu , \gamma ^\sigma ) \nonumber \\ \log (\alpha )\sim & {} \text {Normal}(\log (\alpha )^\mu , \log (\alpha ))^\sigma ) \nonumber \\ \log (\omega )\sim & {} \text {Normal}(\log (\omega )^\mu , \log (\omega ))^\sigma ) \nonumber \\ \log (p)\sim & {} \text {Normal}(\log (p)^\mu , \log (p)^\sigma ) \nonumber \\ \log (r)\sim & {} \text {Normal}(\log (r)^\mu , \log (r)^\sigma ) \nonumber \\ \log (d)\sim & {} \text {Normal}(\log (d)^\mu , \log (d)^\sigma ) \nonumber \\ w\sim & {} \text {Normal}_{(0,1)}(w^\mu , w^\sigma ) \nonumber \end{aligned}$$

We used only weakly informative prior distributions on the group-level parameters to impose minimal constraint on their resulting values:

$$\begin{aligned}{} & {} t_0^\mu \sim \text {Normal}_{(0,\inf )}(.5, .5) \nonumber \\{} & {} A^\mu , B^\mu , V_{\text {target}} \sim \text {Normal}_{(0,\inf )}(2, 2) \nonumber \\{} & {} V_{\text {lure}}^\mu \sim \text {Normal}_{(0,\inf )}(-2, 2) \nonumber \\{} & {} V_0^\mu \sim \text {Normal}_{(0,\inf )}(3.5, 3.5) \nonumber \\{} & {} \eta _{\text {target}}^\mu \sim \text {Normal}_{(0,\inf )}(1,1) \nonumber \\{} & {} \gamma ^\mu \sim \text {Normal}(0, 100) \nonumber \\{} & {} w^\mu \sim \text {Normal}_{(0,1)}(.5, .5) \nonumber \\{} & {} \log (\alpha )^\mu , \log (\omega )^\mu , \log (p)^\mu , \log (d)^\mu , \log (r)^\mu \\{} & {} \quad \sim \text {Normal}(0, 5) \nonumber \\{} & {} t_0^\sigma , A^\sigma , B^\sigma , V^\sigma , V_0^\sigma , \eta _{\text {target}}^\sigma \sim \text {Gamma}(1, 1) \nonumber \\{} & {} \log (\alpha )^\sigma , \log (\omega )^\sigma , \log (p)^\sigma , \log (r)^\sigma , \log (d)^\sigma , \log (\epsilon )^\sigma , w^\sigma \\{} & {} \quad \sim \text {Gamma}(1, 1) \nonumber \\{} & {} \gamma ^\sigma \sim \text {Gamma}(.01, .01) \nonumber \end{aligned}$$

### Details of MCMC parameter estimation

All sampling was conducted using differential evolution Markov chain Monte Carlo (DE-MCMC) techniques (Turner et al., 2013) using Python code written by the first author. For each model, the number of chains was set equal to three times the number of parameters. Each model used a migration phase that occurred every 20 iterations but only between the first 3500 and 5500 iterations. After 20,000 burn-in iterations, one in every ten samples was collected until 1000 samples per chain were collected (10,000 total post burn-in iterations).

A model was considered converged if its Gelman–Rubin (G-R) diagnostic was below 1.2 for all participant and group-level parameters. This criterion was satisfied for all models, with the G-R statistic close to 1.0 in many cases. Some models did not converge on some datasets after the window specified above – these models were re-run using the parameter values from their final iterations – 5000 burn-in iterations were discarded, and then the models were run for an additional 10,000 iterations using the thinning described above.

## Footnotes

1 A reviewer suggested that the same approach could be implemented by incrementing the denominator of the comparison process, which would likely produce equivalent results.

2 We use the term $\eta$ instead of the more popular notation *s* (Brown & Heathcote, 2008) or $s_v$ (Osth et al., 2017) to avoid confusion with the inter-item similarity term *s* above.
