# Supplementary material for: Integrating word-form representations with global similarity computation in recognition memory
Source: Psychon Bull Rev. 2023 Nov 16;31(3):1000–31. doi: 10.3758/s13423-023-02402-2 (PMC11192833; doi:10.3758/s13423-023-02402-2)
Supplement: Supplementary file 1 — (pdf 263 KB) [file 13423_2023_2402_MOESM1_ESM.pdf]

Integrating word-form representations with global similarity computation in recognition memory

Supplementary Materials

Adam F. Osth & Lyulei Zhang

University of Melbourne

Address correspondence to:

Adam Osth (E-mail: adamosth@gmail.com)

Author Note

Abstract

.

Keywords: recognition memory; semantic similarity; semantic space models; linear ballistic accumulator

Integrating word-form representations with global similarity computation in recognition memory

Supplementary Materials

A. Model Fits to the Cortese et al. Dataset

To conserve space, the main text reserved the depictions of the model fit for the winning models. However, fits to the ? (?) data revealed that several of the models made very different predictions for this dataset. Figure 1 illustrates these differences, where we plot the differences between high similarity (HS), moderate similarity (MS), low similarity (low similarity) and very low similarity (VLS) lures, which have a minimum Levenshtein distance to one of the study list items of 1, 2, 3, or greater than or equal to 4, respectively.

To illustrate these differences, each row depicts a class of models contrasted with the winning model, namely the open bigram model. The first row depicts the relative position models (the closed and open bigram models), the second row depicts the absolute position slot code models (slot and both-edges slot code models), the third row depicts the absolute position overlap models, and the fourth row shows the Levenshtein model.

One can see from these plots that the absolute position models generally fail to depict the differences between the high similarity (HS) and medium/low similarity (MS/LS) lures. This contrasts sharply with the generally good fit of the open bigrams model.

Nonetheless, there is little noticeable difference between the open bigram model and its counterpart relative position model - the closed bigram model gets a similarly good fit to the FAR data, but does appear to over-predict the RTs for several of the lure types, which may be why the open bigram model was preferred.

The Levenshtein model generally yields a good account of the FAR, but fails to predict the differences in FAR between the HS lure types, which are distinguished by which letter was missing (the initial, interior, or terminal letter). This is because Levenshtein distance does not consider the position of the transformed letters.

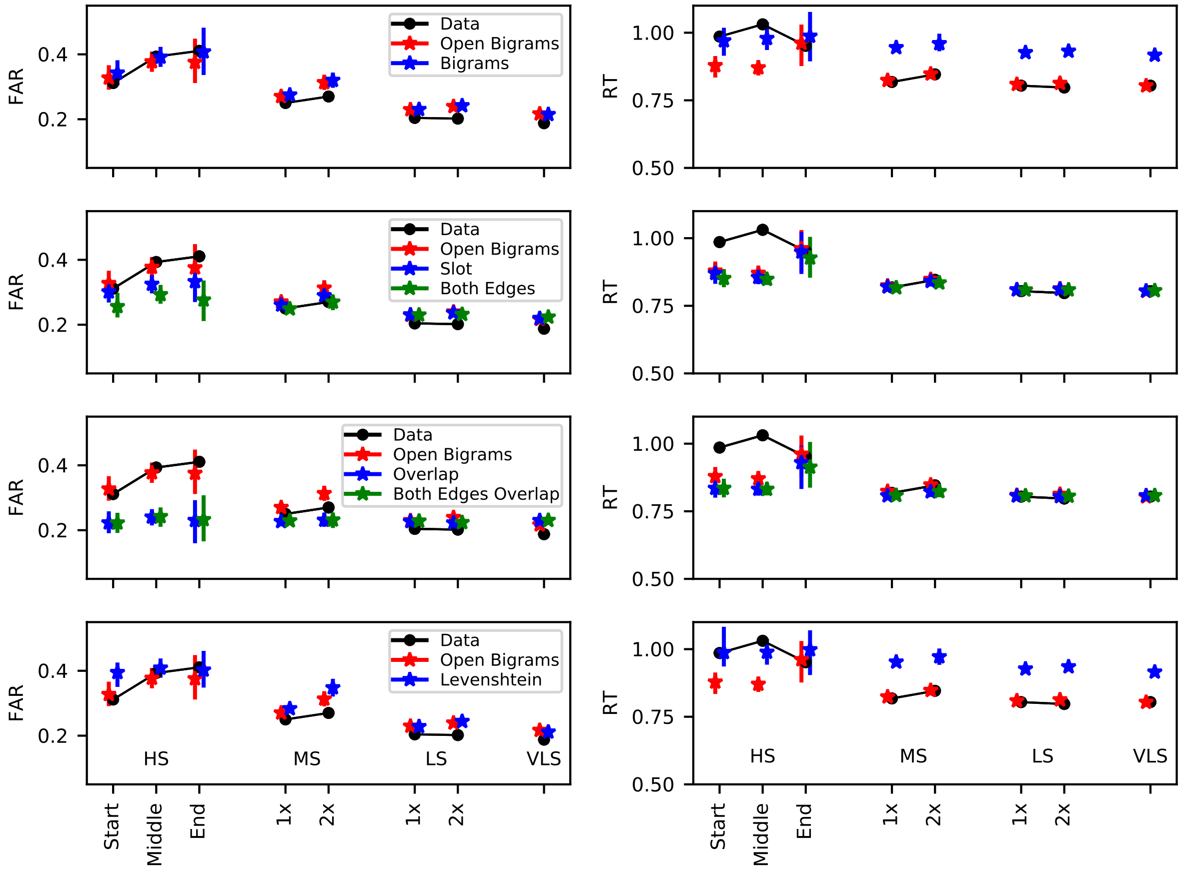

Figure 1. Group-averaged false alarm rates (FAR, left column) and median RTs (right column) to the lure types of varying degrees of similarity for the Cortese et al. (2015) dataset. Error bars depict the 95% highest density interval (HDI). Note: HS = high similarity, MS = medium similarity, LS = low similarity, VLS = very low similarity, 1x = one matching item on the study list, 2x = two matching items on the study list.

B. Individual Differences in the Balance Between Orthographic and Semantic Similarity

In the main text, we reported on the group mean μ estimates of the weight parameter w_o that reflects the weight on orthographic similiarities, while $1 - w_o$ reflects the weight of semantic similarity in the global similarity computation. We found that the group mean parameters generally showed that orthographic representations were at least as consequential as semantic representations and sometimes even exceeded them by a considerable margin, but these estimates varied considerably across datasets in both the

mean and even the uncertainty in the estimates of the group mean parameter w_o^μ.

One possible reason why this parameter estimate varies so considerably is because there is a high degree of variability across participants. We confirmed this possibility here. Figure 3 shows the estimates of the group standard deviation w_o^σ for each dataset. One can see that the two datasets that showed the highest degree of uncertainty in w_o^μ – namely the Criss dataset and the shallow processing condition of the Kiliç et al. dataset – also show very high estimates of w_o^σ.

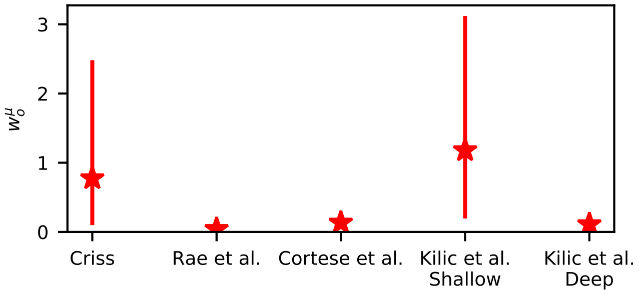

Figure 2. Group standard deviation σ estimates of the weight on orthographic similarity w_o for each dataset. Error bars indicate the 95% highest density interval (HDI).

The high variability across participants in their estimates of w_o is also confirmed by inspection of the individual participant parameters. Figure **??** shows the individual posterior distributions (in gray) for w_o for each dataset (the red posterior distribution shows the group mean w_o^μ). These reveal that there is a considerable degree of uncertainty at the participant level in the estimates of w_o. However, in addition, the aforementioned datasets – namely the Criss dataset and the shallow processing condition of Kiliç et al. – additionally show a considerable amount of variability across participants.

C. LBA Fits to Each Dataset

In the main manuscript, we found that the predictions of the false alarm rates often exceeded those of the data. A question remained as to whether this was driven by the underlying orthographic representations or was due to the LBA architecture itself. The

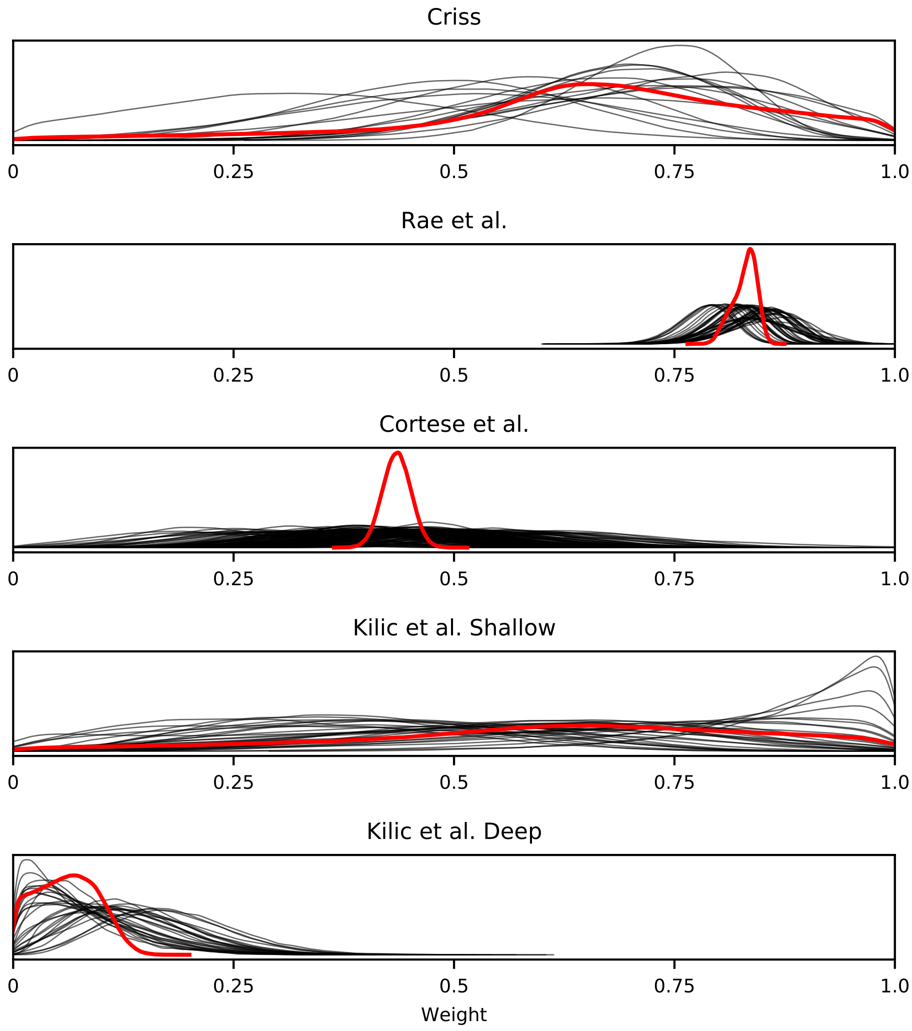

Figure 3. Individual participant posterior distributions of w_o (gray) along with the group mean posterior distribution of $w_o\mu$ (red) for each dataset.

latter is a possibility as the model makes specific predictions about the relationship between RT distributions and choice probabilities – deviations between these relationships that occur in the data force the model to "compromise" in its ability to capture the data.

To address this, we fit the LBA model to each dataset by fixing all of the similarity-related parameters to zero. Essentially, the ability to fit each condition is carried completely by the mean drift rate parameter V_0 that is alotted for each condition. The results can be seen in Figure 4, which show the group-averaged hit rate, false alarm rate

(FAR), and RT quantile predictions for correct and error responses along with the data. These results reveal that the FAR are overpredicted for each dataset. Thus, the overpredicted FAR that are reported in the main text are likely due to the LBA architecture itself and not due to the orthographic representations in the global similarity computation.

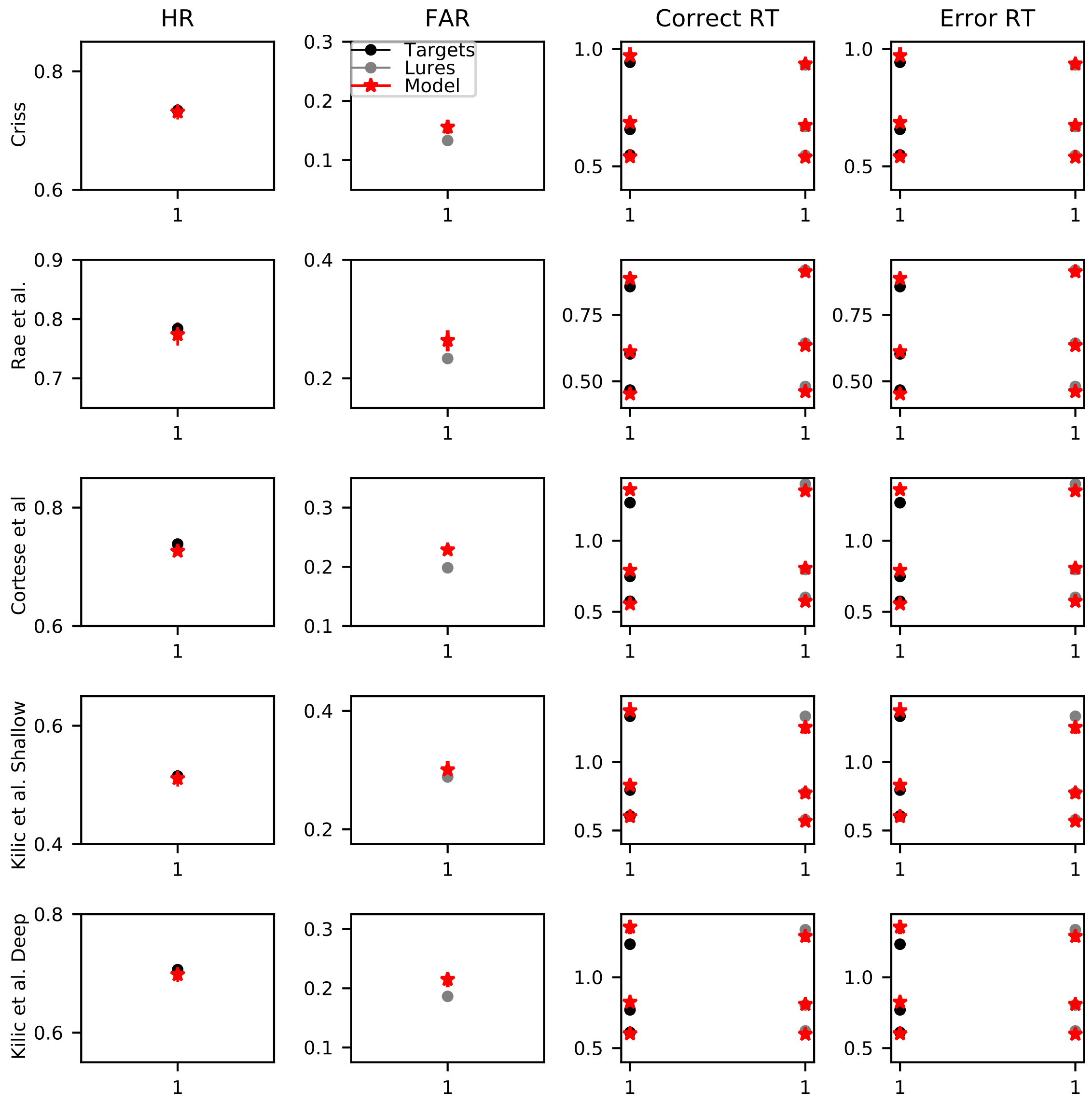

Figure 4. Group-averaged hit rates (HR: first column), false alarm rates (FAR: second column), correct RTs (third column), and error RTs (fourth column) from the data (black) and the winning models (red) of each dataset. RTs are summarized using the .1, .5, and .9 quantile. Error bars depict the 95% highest density interval (HDI).
